# The role and mechanisms of ESM1 in tumors

**DOI:** 10.3389/fonc.2026.1955529

**Published:** 2026-10-23

**Authors:** Siying Zhu, Shuanghua Chen, Zhaoshu Wang, Dan Yuan, Yingzheng Tan

**Affiliations:** 1 Department of Oncology, Zhuzhou Second Hospital, Zhuzhou, Hunan, China; 2 Zhuzhou Clinical College, Jishou University, Jishou, Hunan, China; 3 Hunan Traditional Chinese Medicine College, Zhuzhou, Hunan, China; 4 Clinical Laboratory, Zhuzhou Second Hospital, Zhuzhou, Hunan, China; 5 Department of Infectious Diseases, Zhuzhou Hospital Affiliated to Xiangya School of Medicine, Central South University, Zhuzhou, Hunan, China

**Keywords:** endocrine cell-specific molecule-1 (ESM1), tumor angiogenesis, tumor progression, drug resistance, metabolic reprogramming, immune modulation, tumor suppressor

## Abstract

Endothelial cell-specific molecule-1 (ESM1, also known as endocan), is a sulfated proteoglycan primarily secreted by vascular endothelial cells, although it can also be expressed by tumor cells and stromal cells. Since its overexpression in multiple tumors was systematically confirmed in 2006, ESM1 has become a research hotspot as a tumor biomarker and therapeutic target. This review systematically elaborates on the role of ESM1 in tumors at three levels. First, we analyze the multilayered regulatory mechanisms underlying its upregulation, including transcription factor activation, growth factor and inflammatory signaling, epigenetic modifications, and non-coding RNA regulation, as well as induction by the metabolic microenvironment and pathogen infection. Second, we clarify the dual biological functions of ESM1. In most tumors, ESM1 drives malignant progression through five major dimensions: angiogenesis, malignant behavior and epithelial–mesenchymal transition, metabolic reprogramming, therapeutic resistance, and immune remodeling. Limited evidence suggests that ESM1 may exert antitumor effects in specific contexts, such as prostate cancer, gastric cancer, colon cancer, and non-glycosylated forms of ESM1; however, such evidence is far less abundant than that supporting its pro-tumorigenic role, and its determinants: tumor type, cell source, glycosylation status, disease stage, and experimental model remain unclear. Finally, we evaluate its clinical translational value. As a diagnostic biomarker and prognostic indicator, ESM1 can be detected in various body fluids and is associated with tumor invasion, metastasis, and survival. However, current diagnostic and prognostic evidence is largely retrospective, single-center, and based on non-standardized detection methods. Circulating ESM1 is also confounded by infection, inflammation, tissue damage and bone marrow regeneration, and its cross-cancer diagnostic value has not yet been confirmed. As a therapeutic target, anti-ESM1 strategies show promising prospects for reversing drug resistance and combination therapy, but prospective clinical validation, safety assessment, and evidence for the feasibility of tumor-selective targeting are still lacking. In addition, ESM1 of endothelial origin and ESM1 of tumor or stromal origin may differ in functional interpretation and biomarker interpretation, yet most existing studies have not rigorously distinguished between these sources. In summary, ESM1 is a key molecular node integrating tumor angiogenesis, immune evasion, and therapeutic resistance, and its clinical translational potential as a circulating biomarker and targeted therapy warrants further exploration.

## 1 Introduction

The human *ESM1* gene encodes the ESM1 protein (also known as endocan), a dermatan sulfate proteoglycan that is mainly secreted by vascular endothelial cells, but it can also be expressed by tumor cells and stromal cells. The *ESM1* gene is located on human chromosome 5q11.2, and the mature secreted form consists of a 165-amino acid polypeptide core covalently linked to one dermatan sulfate chain (1, 2). Since its identification as a clone in 1996, early studies on ESM1 have mainly focused on its role in angiogenesis and the regulation of endothelial-specific expression (3, 4). In 2006, a landmark review comprehensively described that *ESM1* is overexpressed in multiple tumors and that its serum levels are closely related to patient prognosis, thereby highlighting for the first time its potential value as a tumor biomarker and therapeutic target (2). Since then, the central role of ESM1 in oncology has rapidly emerged, and numerous studies exploring its mechanisms of action and applications in tumor initiation and progression have rapidly increased. However, ESM1 is heterogeneous in origin, and different cellular sources may affect its functional interpretation and biomarker performance, but most studies have not rigorously distinguished them. In this article, we distinguish endothelial-derived ESM1 from tumor- or stromal-derived ESM1 where possible.

According to the 2022 statistics of the International Agency for Research on Cancer (IARC), there are approximately 20 million new cancer cases and nearly 9.7 million cancer-related deaths worldwide each year, and the number of new cancer cases is projected to surge to 35 million by 2050 (5). Despite continuous advances in treatment strategies, the 5-year survival rate of most patients with advanced cancer has not significantly improved, mainly because of the lack of an ideal target that combines the advantages of early diagnosis, prognostic assessment, and therapeutic intervention (6, 7). Because of its predominant endothelial expression, detectable secretory properties, and multiple roles in processes such as angiogenesis, immunosuppression, and drug resistance, ESM1 is becoming a promising candidate molecule; however, its expression is not tumor-specific and can be affected by inflammation, infection, tissue injury, and bone marrow regeneration (8). Detection methods and cut-off values have also not yet been standardized, and current evidence is mostly preclinical or retrospective. Existing studies are scattered across different cancer types and signaling pathways, and there is still a lack of critical integration of its regulatory networks, context-dependent functions, and clinical translational value. Therefore, this narrative review comprehensively summarizes and critically discusses the context-dependent pro-tumor and tumor-suppressive roles of ESM1 in molecular pathways related to malignant phenotypes, including tumor-mediated angiogenesis, metabolic reprogramming, immunosuppression, and drug resistance, and further evaluates its clinical translational potential as a circulating biomarker and therapeutic target, while emphasizing that most current evidence is preclinical or retrospective and requires prospective validation, thereby providing a theoretical framework for future research and clinical applications of ESM1.

This article is a comprehensive narrative review rather than a systematic review. We searched PubMed, Web of Science, Embase, Scopus, and CNKI from database inception to June 2026. The search strategy combined terms related to ESM1 (“ESM1” OR “endocan” OR “endothelial cell-specific molecule-1”) with terms related to cancer (“cancer” OR “tumor” OR “tumour” OR “neoplasm” OR “carcinoma”). Peer-reviewed original research articles, reviews, and clinical studies published in English or Chinese were included. Conference abstracts, duplicate publications, articles without available full text, and studies unrelated to cancer were excluded. Two authors independently screened titles and abstracts, and disagreements were resolved through discussion; reference lists of key reviews were also manually checked.

## 2 Upstream drivers and regulatory networks of *ESM1* overexpression

### 2.1 Transcriptional activation by tissue-specific transcription factors and hypoxia

The high expression of *ESM1* in tumors is regulated by a two-tiered transcription factor network that is both tumor-type-specific and universally responsive to the tumor microenvironment.

Transcription Factor Activation Network: At the tumor-type-specific level, the *ESM1* promoter can be directly activated by specific transcription factors in different tumor microenvironments. Existing studies have identified these upstream regulatory factors in several cancer types using various experimental approaches. For example, He et al. demonstrated using a dual-luciferase reporter assay and chromatin immunoprecipitation experiments that SPI1 directly binds to the *ESM1* promoter in endometrial cancer (9). Similarly, HOXD4 in gliomas (10), AP-1 in head and neck squamous cell carcinoma (HNSCC) (11) and BCL6 in hepatocellular carcinoma (HCC) have been implicated (12). Furthermore, when COL5A1, a component of the extracellular matrix in gliomas, is overexpressed, it may induce upregulation of *ESM1* by activating the transcription factor PPRC1 (13). In colorectal cancer (CRC), *ESM1* may also be regulated by upstream transcription factors (14). These studies indicate that *ESM1* responds to oncogenic signals in different tissues, and differences in these upstream factors may be one of the reasons for its varying functions across different cancers. From a clinical perspective, interfering with the binding of key transcription factors (such as SPI1 and AP-1) to the *ESM1* promoter in specific cancer types holds promise for achieving precise transcriptional repression. This approach could enable the specific silencing of *ESM1* in specific cancer types, thereby providing new insights into the development of anticancer drugs.

Hypoxia-Induced Activation: Besides, hypoxia, a microenvironmental feature common to many solid tumors, can universally and efficiently induce *ESM1* expression via the transcription factor hypoxia-inducible factor 1-alpha (HIF-1α). The Gu team found that in non-small cell lung cancer (NSCLC), chronic intermittent hypoxia directly activates *ESM1* transcription by causing HIF-1α to bind to the HRE in the *ESM1* promoter (15). In CRC and ovarian cancers (OC), hypoxia can also induce HIF-1α to directly activate the transcription of *ESM1* (16, 17). The HIF-1α-*ESM1* axis is activated in response to hypoxia in NSCLC, OC, and CRC, suggesting that this pathway may be universal across cancers. Therefore, it holds promise as a broad-spectrum therapeutic target.

The two-tier regulatory model described above explains the high *ESM1* expression in tumors. However, the interaction between these two mechanisms at the promoter level remains unclear, limiting the development of upstream-targeting strategies. Elucidating their competitive or synergistic mechanisms will be key to developing precision interventions in the future.

### 2.2 Induction by angiogenic growth factors and inflammatory cytokines

Angiogenic Factors: Angiogenesis-promoting growth factors are key regulators of *ESM1* expression. Among these, the role of vascular endothelial growth factor (VEGF) is the most well established. In models such as clear cell renal cell carcinoma (ccRCC), VEGF can directly upregulate *ESM1* transcription and synthesis through signaling pathways such as PI3K and can also directly stimulate endothelial cells to secrete ESM1 (18, 19). In bladder cancer (BCa), VEGFA exerts a similar effect through the vascular endothelial growth factor receptor 2 (VEGFR2) phosphorylation cascade (20). In addition, a subset of fallopian tube epithelial cells with high VEGF expression in OC can interact with ESM1-positive endothelial cells (apical cells), suggesting that VEGF may indirectly promote elevated ESM1 levels by inducing the phenotypic transformation of endothelial cells (21). In CRC, VEGFA induces apical endothelial cell-specific expression of *ESM1* via the kinase insert domain receptor (KDR) signaling pathway, thereby forming a VEGFA-KDR-*ESM1* positive feedback loop (22). In addition to VEGF, fibroblast growth factor 2 (FGF2) has been shown to upregulate *ESM1* expression in prolactinomas and gliomas (23, 24). In addition, nerve growth factor receptor (NGFR), epidermal growth factor (EGF), and FGF2 signaling play similar regulatory roles in Oral Squamous Cell Carcinoma (OSCC) and malignant pleural mesothelioma (MPM) (25, 26). Thus, ESM1 is a common target for various pro-angiogenic signals.

Inflammatory Cytokines: Inflammatory cytokines are another important class of regulatory factors. Tumor necrosis factor-alpha (TNF-α), a classic inflammatory mediator, is also a potent transcriptional activator of *ESM1*; in a bevacizumab-resistant model, it significantly increased *ESM1* mRNA levels (27). In gliomas, VEGFA and transforming growth factor beta 2 (TGFβ2) can act synergistically to drive the upregulation of genes associated with vascular abnormalities, such as *ESM1* (28).

Elevated ESM1 levels are regulated by both angiogenic factors and inflammatory cytokines. As upstream regulators of *ESM1*, such as VEGF and TNF-α, can be secreted by tumor cells or immune cells, the high expression of ESM1 in tumors reflects an integrated response to signals from multiple cellular sources in the microenvironment. At the same time, this suggests that ESM1 may serve as a hub linking the two major hallmarks of cancer: angiogenesis and inflammation. When these two types of signals act in concert, *ESM1* expression may be further amplified, driving malignant tumor progression. Therefore, targeting these upstream signals or ESM1 may be promising for breaking this vicious cycle.

### 2.3 Epigenetic modifications and non-coding RNA-mediated regulation

Epigenetic modifications and post-transcriptional regulation are other key factors influencing *ESM1* expression. Studies have shown that in triple-negative breast cancer (TNBC), the overexpression of *ESM1* in the more aggressive brain metastasis cell line (MDA-MB-231BR) is associated with low DNA methylation in the upstream region of the gene (29). In addition, histone modifications also play a role; in HCC, elevated levels of histone lactylation (H3K9la and H3K56la) can directly bind to the *ESM1* promoter region and promote its transcription (30).

In non-coding RNA-mediated regulation, various RNA molecules influence *ESM1* expression via competitive binding mechanisms. In breast cancer (BC), the circular RNA hsa_circRNA_102908 can bind to miR-410-3p, thereby lifting its inhibitory effect on *ESM1* and upregulating *ESM1* (31). Similarly, the lncRNA SNHG14 in BCa and lncRNA HULC in glioma also bind to miRNAs, such as miR-211-3p, via the ceRNA mechanism, thereby upregulating *ESM1* (32, 33). In addition, the reduced expression of miR-9-3p derived from exosomes in BCa weakens the inhibitory effect on *ESM1*, leading to elevated *ESM1* levels (34). In addition to non-coding RNAs, protein-RNA interactions play a role in the post-transcriptional regulation of *ESM1*. In melanoma, LIMK2 kinase activates the RNA-binding protein G3BP1 through phosphorylation; G3BP1 subsequently stabilizes *ESM1* mRNA, leading to elevated *ESM1* levels (35), indicating that the regulation of *ESM1* extends to mRNA stability.

As described above—from DNA methylation and histone modifications to competitive binding of non-coding RNAs and regulation of mRNA stability—*ESM1* is subject to multiple layers of fine-tuned regulation at the epigenetic and post-transcriptional levels, further highlighting the complexity of its expression regulatory network.

### 2.4 Metabolic microenvironment and pathogen-driven upregulation

Several metabolites have been shown to be effective inducers of *ESM1* in the metabolic microenvironment. In OC, lactate can directly upregulate *ESM1* expression (36, 37). In addition to lactate, high cholesterol levels have been shown to indirectly upregulate *ESM1*. Gao et al. found that cholesterol enhances *ESM1* mRNA stability via the m6A reader protein IGF2BP3, thereby forming an IGF2BP3/*ESM1*/KLF10/BECN1 regulatory loop (38). Similar metabolic signals include deoxycholic acid from refluxed bile in esophageal cancer (EC) and elevated *ESM1* levels following melatonin treatment in gastric adenocarcinoma (39, 40). In addition, butyrate in CRC leads to the upregulation of *ESM1* by inhibiting the phosphorylation of the MAPK/ERK2 pathway (41). In TNBC, extracellular ATP activates P2Y2R, thereby initiating the JNK/p38 MAPK cascade, which ultimately drives *ESM1* expression (42).

In addition to metabolic signals, pathogen infection can drive the upregulation of *ESM1.* In cervical cancer, the E6/E7 oncoproteins of high-risk HPV downregulate miR-205-5p, which targets *ESM1*, thereby lifting its suppression of *ESM1* mRNA and significantly upregulating *ESM1* (43). In malignant gliomas, HCMV infection is positively correlated with ESM1 levels in tumor tissue and patient serum, and *in vitro* experiments have confirmed that HCMV infection can directly induce *ESM1* expression (44). In nasopharyngeal carcinoma (NPC), the Epstein-Barr virus latent membrane protein 1 (LMP1) activates the NF‐κB, MEK-ERK, and JNK pathways through its CTAR1/CTAR2 domains, thereby continuously upregulating *ESM1* expression (45). In addition, in patients with acute myeloid leukemia (AML), concurrent bacterial infections can cause a sudden increase in serum ESM1 levels (46).

ESM1 serves as a molecular hub that is activated by multiple signaling pathways. Whether it is the accumulation of lactate or bile acids within a tumor or exogenous viral or bacterial infections, these various stimuli ultimately lead to the upregulation of *ESM1* expression. This suggests that the elevation of *ESM1* is not coincidental but is likely an adaptive response program activated by cells to adapt to their environment and maintain survival in response to disturbances in the metabolic microenvironment or infection. Therefore, targeting these upstream inducing factors—such as modulating the metabolic microenvironment or administering antiviral/anti-infective therapies—may indirectly suppress *ESM1* expression, offering a potential new approach for combination therapy to improve the tumor microenvironment and enhance the efficacy of existing treatments. (**Figure 1**)

**Figure 1:**
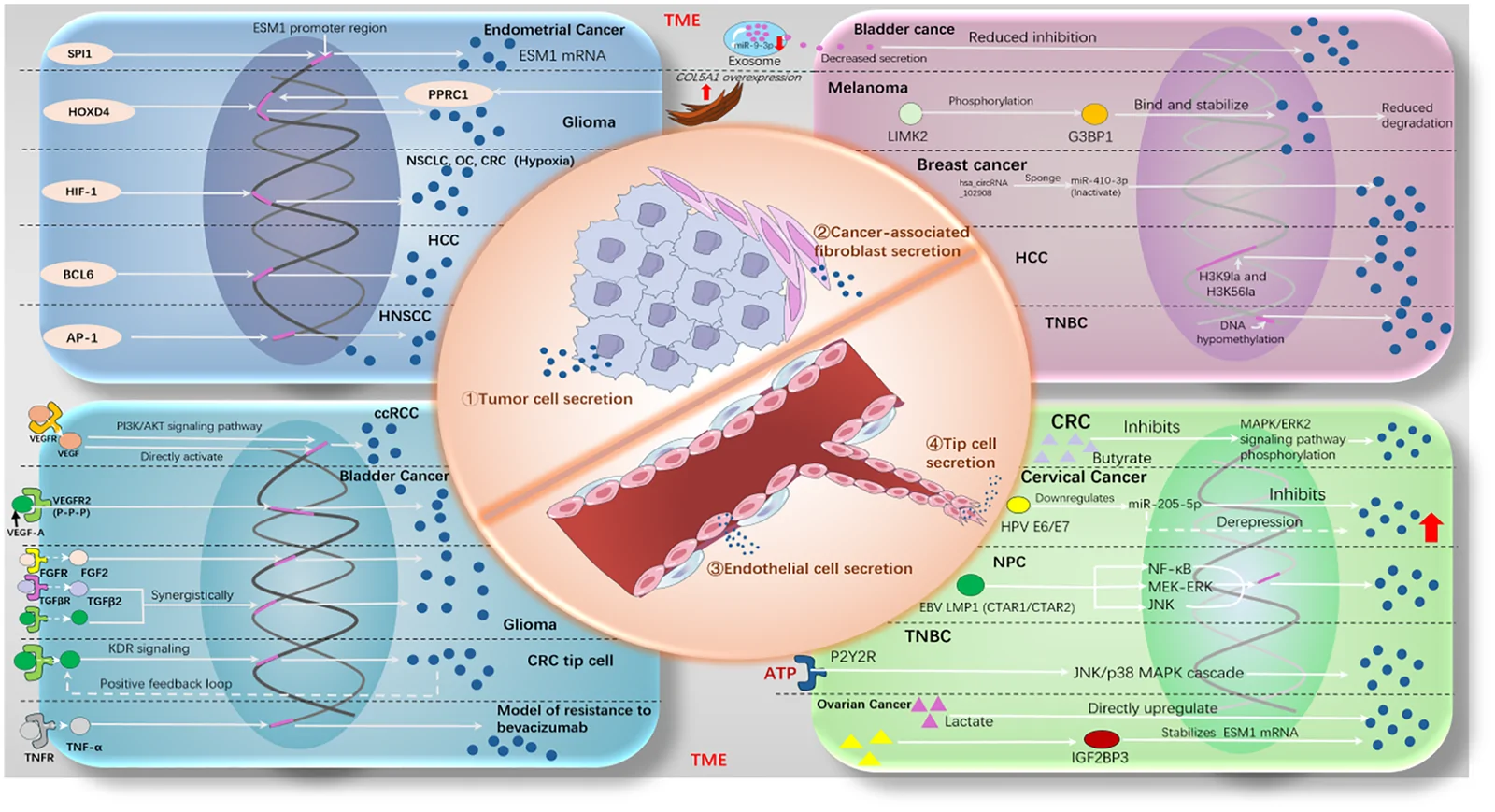
Upstream regulatory mechanisms of *ESM1* in the tumor microenvironment. ESM1 is secreted by tumor cells, cancer-associated fibroblasts, endothelial cells, and tip cells. Its expression is regulated at multiple levels, including tissue-specific transcription factors and hypoxia, angiogenic growth factors and inflammatory cytokines, epigenetic modifications and non-coding RNAs, and metabolic and pathogen-derived signals. See the main text for detailed mechanisms.

## 3 Oncogenic functions and signaling mechanisms of ESM1 in tumor progression

### 3.1 Promotion of tumor angiogenesis via multiple signaling axes

Angiogenesis is a crucial step in tumor growth and metastasis. Preclinical studies suggest that ESM1 may act not only as an upstream initiator of signaling pathways but also as a key node in positive feedback loops. It has also been reported to mediate intercellular communication through protein interactions and to amplify pro-angiogenic effects through post-translational modifications, potentially forming a multi-layer regulatory network that promotes angiogenesis in preclinical models. However, many of these conclusions are based on a limited number of cell lines and preclinical models, and some pathway associations are derived from bioinformatics or Gene Set Enrichment Analysis (GSEA) analyses rather than direct functional validation. These findings should therefore be interpreted as preclinical and hypothesis-generating, and independent *in vivo* and clinical validation is still required.

As a signaling initiator, ESM1 has been reported to activate multiple canonical signaling pathways in preclinical models. Preclinical evidence based on cell and animal models indicates that in CRC, ESM1 activates the PI3K/Akt/mTOR pathway and upregulates angiogenesis-related proteins such as Cyclin D1, VEGF, MMPs, and HIF-1α. This activation subsequently promotes tumor angiogenesis and accelerates tumor progression, whereas treatment with a PI3K inhibitor attenuates these effects (47). A similar phenomenon has been observed in glioma, in which the long non-coding RNA HULC regulates *ESM1* expression through the same PI3K/Akt/mTOR axis to promote angiogenesis. In addition, ESM1 interacts with receptor tyrosine kinase (RTK) signaling (33). However, this mechanism is mainly based on cell line experiments and still requires further validation in *in vivo* models. In gastric cancer (GC), ESM1 secreted by tumor cells binds to c-Met on the membrane of vascular endothelial cells to activate the MAPK/ERK pathway, upregulating VEGFA, HIF-1α, and MMP9, thereby significantly promoting angiogenesis in GC (48). This study also revealed a bidirectional relationship: VEGFA can stimulate *ESM1* expression via VEGFR-1 phosphorylation, whereas ESM1 displaces VEGFA165 by binding to fibronectin and enhances its bioavailability, thereby forming a positive feedback loop (48). In cervical squamous cell carcinoma (CSCC), ESM1 was reported to activate the VEGFα/VEGFR2–ERK1/2 axis. This effect may synergize with HIF-1α to drive angiogenesis (49). These conclusions were mainly based on immunohistochemistry (IHC), public databases, and SiHa/ME-180 cell experiments. However, these mechanistic conclusions are mainly derived from GSEA and *in vitro* experiments and still require validation in *in vivo* models and blood samples. In OC, functional experiments suggest that ESM1 drives angiogenesis via the Akt/mTOR pathway, and its knockdown reduced VEGF levels and inhibited endothelial tube formation (21). In lung adenocarcinoma (LUAD), ESM1 has been reported to activate the AKT signaling pathway to upregulate FASN and SCD1, promoting fatty acid synthesis in both tumor cells and endothelial cells, thereby driving angiogenesis in a paracrine manner (50). In kidney renal clear cell carcinoma (KIRC), ESM1 simultaneously activates the Akt/mTOR and Ras pathways, and its paracrine function can directly enhance human umbilical vein endothelial cell (HUVEC) proliferation, migration, and tube formation (51). This study reported *in vitro* and *in vivo* evidence, but the mechanistic conclusions remain primarily based on KIRC cell lines and endothelial cell functional experiments, and independent validation in additional *in vivo* models and clinical samples is still required.

In addition to activating a single pathway, ESM1 also has been reported to participate in complex positive feedback loops that amplify angiogenic signaling. In OC cells, endothelial cells, and animal models, ESM1 directly binds to ANGPTL4 (confirmed by Co-IP and molecular docking); on the one hand, this stabilizes the binding of ANGPTL4 to LPL and drives lipid metabolic reprogramming, while on the other hand, it interferes with the binding of ANGPTL4 to integrin α5β1 and VE-cadherin, ultimately promoting functional angiogenesis (52). However, the effects of ANGPTL4 on angiogenesis are context-dependent, and its interaction with ESM1 may be a key determinant of the direction of its angiogenic effects. These conclusions are mainly based on preclinical models and require further functional and clinical validation.

ESM1 may also regulate angiogenesis by mediating intercellular communication. In BCa, single-cell sequencing identified an MMP11+ myofibroblast-like cancer-associated fibroblast (CAF) subset that gradually accumulated during tumor progression and was associated with poor prognosis. After induction by tumor cell-derived BMP2, this subset, on the one hand, secreted WNT5A to enhance the migration of ESM1+ tip endothelial cells via the WNT5A–MCAM axis, and on the other hand, recruited SPP1+ macrophages via CCL11/CCL2 to secrete VEGFA, collectively activating the pro-angiogenic activity of ESM1+ endothelial cells (53). In mouse experiments, BMP2 inhibitors inhibited tumor angiogenesis; however, the drug used, dorsomorphin, has off-target effects. Given the limited available evidence, the relevant conclusions should be regarded as exploratory findings (53). Furthermore, in bevacizumab-resistant TNBC cells and mouse models, *ESM1* is transcriptionally upregulated by the TNFα–NF‐κB–RelB axis; it simultaneously upregulates VEGF and DLL4 to drive functional angiogenesis and metastasis, and this process can be reversed by an anti-ESM1 antibody (27). Similarly, in preclinical models of bevacizumab-resistant CRC and TNBC, ESM1 acts as an upstream regulator of DLL4, activates Notch signaling, and promotes MMP9 expression, synergistically driving angiogenesis and metastasis (54). However, these findings are based mainly on preclinical models and require further validation in clinical settings.

Post-translational modifications have been reported to further enhance the pro-angiogenic function of ESM1. In bevacizumab-resistant OC cell lines and mouse models, TRIM28 promotes SUMOylation of ESM1, stabilizing the ESM1 protein by inhibiting its proteasomal degradation. Secreted ESM1 subsequently activates the ITGB1/FAK pathway to drive angiogenesis and drug resistance (55). Furthermore, in OC, by integrating patient sample lactylomes, cell lines, patient-derived xenograft (PDX), zebrafish, and chorioallantoic membrane (CAM) models, AARS1-mediated lactylation of enolase 1 (ENO1) at K71 enhances lactate synthesis and promotes histone lactylation (H3K9la and H3K14la), thereby transcriptionally upregulating *ESM1* at the epigenetic level. Secreted ESM1 stabilizes Yin Yang 1 (YY1) in endothelial cells by competitively inhibiting Smurf2-mediated ubiquitination, and subsequently recruits E1A binding protein p300 (EP300) to induce H3K27 acetylation at the B-cell lymphoma 2-related protein A1 (BCL2A1) promoter, ultimately enhancing endothelial cell survival and angiogenesis (56). However, these mechanistic conclusions are mainly derived from cell lines and animal models, and independent validation in human samples and clinical cohorts is still lacking.

### 3.2 Driving tumor malignancy and epithelial–mesenchymal transition

In addition to driving tumor angiogenesis, ESM1 has been reported to regulate tumor-cell malignant behavior in several experimental models, although the cellular source of ESM1 differs among studies and is not always clearly defined. Studies have shown that ESM1 may achieve these functions by activating several key signaling pathways, including the classical PI3K/Akt/mTOR, NF‐κB, Wnt/β-catenin, and RTK/Ras signaling cascades, as well as several non-classical signaling networks. However, the currently available evidence is mainly derived from cell lines, animal models, and bioinformatics analyses, and clinical validation remains limited; most studies have not strictly distinguished the cellular source of ESM1, and the cellular sources, experimental systems, and tumor types used vary considerably across studies, so the conclusions still require independent validation. In particular, several findings are based on only one or two cell lines, and *in vivo* xenograft validation among the tumor-suppressive studies is currently limited mainly to prostate cancer. In addition, some pathway associations derived from GSEA or bioinformatics analyses are correlative and do not by themselves establish functional pathway activation.

#### 3.2.1 The PI3K/Akt/mTOR signaling axis

The PI3K/Akt/mTOR pathway is one of the key pathways most frequently reported in ESM1-associated malignant phenotypes, and its regulatory mechanisms may involve multiple layers of complexity.

Secreted ESM1 from GC cells has been reported to enhance EGFR/HER3 dimerization and activate PI3K, leading to elevated p-Akt levels, epithelial–mesenchymal transition (EMT), and increased migration and invasion. The signal peptide-deleted mutant ESM1-19del loses this tumor-promoting effect, suggesting that secreted ESM1 is essential for its function. This study also found that ESM1 has functional synergy with ANGPT2, together promoting cancer progression through Akt signaling (57). However, these conclusions are mainly derived from GC cell lines, and independent validation in additional models and *in vivo* systems is still required. In TNBC cell experiments and xenograft models, ESM1 has been reported to upregulate Cyclin D1 expression by activating Akt-dependent NF‐κB signaling, thereby promoting cell cycle progression and proliferation (58), indicating that this pathway may regulate both EMT and proliferation.

At the level of transcriptional regulation, ESM1 in cervical cancer cells has been reported to directly regulate the core transcriptional program of EMT by activating the ZEB1/PI3K/AKT axis (59). Based on RNA sequencing and functional experiments, SYT13 was identified as a key downstream effector molecule of ESM1 that mediates cell migration and invasion; however, this conclusion is mainly derived from cervical cancer cells and xenograft models (60). Both pathways suggest activation of EMT; however, it cannot yet be inferred that ESM1 drives malignant phenotypes through the same mechanism in all cervical cancers. These findings are based on a limited number of cervical cancer cell lines, and independent validation in additional models is required.

At the level of paracrine/autocrine networks, ESM1 secreted by ccRCC/KIRC tumor cells has been reported to simultaneously activate the Akt/mTOR and Ras signaling pathways, promoting the invasive capacity of tumor cells and the tube formation capacity of endothelial cells through autocrine and paracrine mechanisms (51), suggesting that the ESM1-Akt axis may exert multifunctional roles in intercellular communication among different cell types. However, this evidence is mainly based on KIRC cell lines and endothelial functional experiments, and further *in vivo* validation is needed.

Furthermore, in CRC, Olink proteomic analysis based on tissues from 52 patients identified ESM1 as one of the most significantly altered proteins, suggesting an association with the PI3K/AKT oncogenic pathway; however, this study was a tissue proteomic association analysis and did not directly validate the function of ESM1, and functional experiments are still required (61). In summary, in models including GC, TNBC, cervical cancer, and ccRCC/KIRC, ESM1 has been reported to participate in the regulation of EMT, proliferation, and invasion through the PI3K/Akt/mTOR pathway; however, given the varying experimental systems and cellular sources across studies, the universality of this pathway still requires independent validation. These findings are based primarily on cell lines and xenograft models, and their clinical relevance remains to be validated.

#### 3.2.2 The NF‐κB survival program

ESM1 has been reported to play a key role in maintaining tumor cell survival and resistance to apoptosis by regulating the NF‐κB pathway. Among solid tumors, CRC cell experiments showed that ESM1 may activate NF‐κB through an Akt-dependent mechanism, promoting cell survival and cell cycle progression (via Cyclin D1) and enhancing migration, invasion, and EMT. In COLO205 and HCT-116 cells, *ESM1* knockdown or overexpression correspondingly altered NF‐κB pathway activity (62). Furthermore, HCC cell experiments suggested that *ESM1* silencing impaired cell survival by inhibiting the NF‐κB pathway and induced cell cycle arrest by suppressing Cyclin D1 via PTEN induction (63). In hematologic malignancies, experiments using U937 and K562 leukemia cells showed that knockdown of *ESM1* in leukemia cells induced G0/G1 cell cycle arrest, downregulated Cyclin D1, and promoted apoptosis by inhibiting NF‐κB activity (64). These conclusions are based on a limited number of cell lines, and independent validation in additional models and clinical samples is still needed.

In summary, these preclinical studies suggest that the ESM1–NF‐κB axis may participate in cell survival in solid tumors and hematologic malignancies; however, these conclusions have not yet been validated in clinical settings. Moreover, most evidence is derived from a small number of cell lines, and *in vivo* validation remains limited.

#### 3.2.3 Wnt/β-catenin signaling pathway

Activation of the Wnt/β-catenin pathway is a classic oncogenic pathway that regulates tumor cell proliferation, survival, and invasion (65). ESM1 has been reported to participate in the regulation of this pathway through both indirect and direct mechanisms of action.

At the level of indirect activation, analyses of OC cells and patient samples showed that lactate-induced ESM1 may activate the Wnt/β-catenin pathway by upregulating SCD1 expression, thereby impairing CD8+ T cell function and promoting malignant behaviors such as immune evasion, proliferation, and metastasis of tumor cells (36). However, this conclusion is mainly based on OC cell lines and patient sample correlation analyses, and functional validation *in vivo* is still required.

At the level of direct interaction, the nuclear form of ESM1 in prostate cancer has been reported to directly interact with the ARM domain of β-catenin, stabilizing the β-catenin/TCF4 transcriptional complex, enhancing the transcription of Wnt target genes, and promoting the maintenance of tumor stem cell characteristics and metastasis (66), suggesting a new function of ESM1 as a transcriptional coactivator. This evidence is mainly derived from prostate cancer cells and *in vivo* models and still requires validation in additional tumor types.

In melanoma tissues and mouse models, as well as in ccRCC tissues and cell experiments, high ESM1 expression was also associated with activation of the Wnt/β-catenin signaling pathway, thereby possibly promoting tumor proliferation and progression (67, 68). However, most of these studies are based on bioinformatics, IHC/WB (Western blot), and correlation analyses and still require functional validation; therefore, it cannot be concluded that this pathway is universal in ESM1 function. These results remain preclinical and require further *in vivo* and clinical validation.

#### 3.2.4 The RTK/Ras signaling network

A bidirectional regulatory relationship has been proposed between ESM1 and RTK signaling: ESM1 can act as a downstream effector of RTK signaling and also as an upstream regulator that enhances RTK activity. In an overexpression model of endothelial-derived ESM1, ESM1 relies on its glycosylation domain and phenylalanine-rich region to induce solid tumor formation by binding to and enhancing HGF activity; in HEK293 overexpression and severe combined immunodeficient (SCID) mouse models, a monoclonal antibody against the F region significantly reduced tumor growth (69). This study suggests that endothelial-derived ESM1 may promote tumor formation; however, the model used was an engineered overexpression system. Therefore, these findings require validation in more physiological models.

In radioresistant TNBC cells, ESM1 was shown to be upregulated through VEGFR2, which integrates stress signals (e.g., ATP) with growth factor receptor signaling. This may confer acquired radioresistance and enhanced invasiveness (42). These mechanistic inferences are largely derived from *in vitro* and animal studies and should be interpreted with caution.

#### 3.2.5 Non-classical signaling axes

In addition to the classic pathways mentioned above, ESM1 may expand its functional network through diverse signaling pathways, including JAK/STAT, chemokine regulation, and Notch.

At the level of signal transduction, based on The Cancer Genome Atlas (TCGA)/GSEA analysis and functional experiments in EC cells, high *ESM1* expression was closely associated with JAK signaling pathway activity; knockdown of *ESM1* significantly inhibited tumor cell proliferation and migration and was accompanied by decreased JAK1 protein levels, suggesting that the ESM1–JAK axis is a key pathway underlying its oncogenic behavior (70). However, these conclusions are based on GSEA and a limited number of cell lines and still require validation in additional independent cohorts and *in vivo* experiments. Moreover, GSEA-derived associations should be interpreted as correlative rather than as direct evidence of pathway activation.

At the level of microenvironmental interactions, knockdown of *ESM1* in prostate cancer PC-3 cells significantly inhibited cell migration, accompanied by downregulation of the angiogenic chemokine CXCL3 (71), thereby preliminarily suggesting a functional link between ESM1 and CXCL3 regulation. This study involved only a single cell line experiment, and the mechanism remains unclear. Therefore, this finding should be regarded as exploratory.

At the level of fine cell cycle regulation, analyses of adrenocortical carcinoma (ACC) samples/databases and experiments in SW13 cells showed that ESM1 may activate the DLL4–Notch signaling pathway and regulate the key downstream cell cycle effectors CDK1 and p21, thereby promoting tumor cells through the G2/M transition and promoting proliferation (72). This conclusion is mainly based on ACC cell lines and remains to be validated in *in vivo* models. In addition, because part of the evidence is derived from database analyses, the possibility of correlation rather than causation cannot be excluded.

At the transcriptional and epigenetic levels, endothelial cell-secreted ESM1 has been reported to directly bind to and activate the PDGFRA receptor on the surface of glioblastoma (GBM) cells, and then, through downstream signaling, enhances chromatin accessibility at the Myc gene promoter region and stably upregulates Myc protein expression, thereby significantly enhancing angiogenesis, proliferation, and migration. In *ESM1*-knockout mouse models, the survival benefit of the PDGFRA inhibitor ponatinib depended on *ESM1* expression status (73). This study supports that endothelial-derived ESM1 acts on GBM cells via the PDGFRA–Myc axis; however, the structural basis of *ESM1* binding to PDGFRA remains unclear. Moreover, these conclusions are mainly derived from cell lines and animal models, and independent validation in human samples and prospective clinical cohorts is still lacking.

### 3.3 Mediation of chemotherapy, targeted therapy, and radiotherapy resistance

Preclinical studies suggest that ESM1 may participate in tumor cell adaptation to therapeutic stress and may be associated with resistance to chemotherapy, targeted therapy, and radiotherapy in some models. However, the currently available evidence is mainly derived from cell and animal models, and clinical evidence directly demonstrating its role in driving resistance remains limited. In addition, many of these findings are based on a limited number of cell lines and *in vitro* assays, and no ESM1-targeted strategy has yet entered clinical trials. Therefore, the following observations should be regarded as preclinical and hypothesis-generating rather than clinically established.

#### 3.3.1 Chemotherapy resistance

In OC cell models, multiple studies suggest that ESM1 may be involved in chemotherapy resistance. Specifically, in OC cells, the ESM1–SCD1 axis has been reported to upregulate P-glycoprotein (P-gp) and to be associated with platinum resistance; this study also suggested that the ESM1–SCD1 axis may participate in immune evasion, but its clinical relevance has not yet been validated (36). Another mechanism involves ESM1 in OC cells inhibiting the expression of lncRNA GAS5, thereby relieving the binding of GAS5 to miR-23a-3p, which enables miR-23a-3p to target and inhibit PTEN, potentially activating the PI3K/AKT pathway. Functional experiments suggest that this promotes cisplatin resistance. However, this conclusion is based on OC cell lines and *in vitro* functional experiments and remains to be validated in *in vivo* models (74). Furthermore, in OC cells, ESM1 has been reported to bind to ERBB2 to activate the FAK/SRC and NF‐κB signaling pathways, leading to upregulated HSPB1 expression, thereby inhibiting ferroptosis and enhancing cisplatin resistance (75). This study was primarily based on cell experiments and public database analyses, and clinical validation remains limited. In addition, these conclusions are mainly based on cell lines and *in vitro* functional assays, and independent *in vivo* validation is still lacking. They should therefore be interpreted as preliminary mechanistic findings rather than established mechanisms of clinical resistance.

#### 3.3.2 Drug resistance to targeted therapy

Preclinical evidence suggests that high *ESM1* expression may be associated with poor response to anti-angiogenic therapies such as bevacizumab. In bevacizumab-resistant TNBC and CRC cells and mouse models, ESM1 has been reported to participate in MMP9-, VEGF-, and DLL4-mediated angiogenesis and metastasis and to be associated with lung metastasis and a resistant phenotype (27, 54). Mechanistically, ESM1 has been proposed to link inflammatory signaling to aberrant angiogenesis and may serve as a potential node for reversing resistance; in bevacizumab-resistant mouse models, the combination of bevacizumab with an anti-ESM1 monoclonal antibody was reported to improve the therapeutic response (54). However, these findings are based on a limited number of cell lines and xenograft models, and independent validation in additional models and clinical samples is still required. In HCC, based on cell lines, patient-derived xenograft models, and retrospective clinical tissue microarray analyses, ESM1 and ANGPTL4 have been reported to form a positive feedback loop that enhances the stability of FASN by inhibiting its ubiquitin-mediated degradation. This may promote triolein synthesis, which subsequently may activate the NF‐κB/IL-17 pathway in HCC cells and induces CD99 expression in endothelial cells, thereby contributing to angiogenesis and bevacizumab resistance. In addition, clinical data (tissue microarray) indicate that in HCC patients resistant to bevacizumab, ESM1 and ANGPTL4 expression are elevated and may be associated with poor prognosis (76). However, this was a retrospective analysis, and a causal relationship has not been established. In drug-resistant OC cell lines and mouse models, TRIM28 has been reported to act as an upstream regulator that stabilizes ESM1 protein by promoting its SUMOylation, thereby inhibiting its proteasomal degradation. ESM1 secreted by drug-resistant OC cells subsequently has been reported to activate the ITGB1/FAK axis, contributing to angiogenesis and bevacizumab resistance. In mouse models of OC, TRIM28 overexpression has been demonstrated to promote angiogenesis and bevacizumab resistance through ESM1-mediated ITGB1/FAK activation (55). These findings remain preclinical, and no ESM1-targeted strategy has yet entered clinical trials. Therefore, any clinical translation remains speculative, and prospective clinical validation is required before clinical application can be considered.

#### 3.3.3 Other types of drug resistance

In addition to the conventional therapeutic modalities described above, preclinical evidence suggests that ESM1 may be associated with radiotherapy resistance. As previously mentioned, in radioresistant BC and GBM cells and mouse models, high *ESM1* expression has been reported to be associated with radiotherapy resistance (73, 77). However, these observations are mainly derived from a limited number of radioresistant cell lines and animal models, and whether ESM1 directly drives radiotherapy resistance remains to be established. The evidence should therefore be regarded as correlative and preliminary rather than causal. In addition, based on the analysis of clinical samples from only 25 patients with prolactinoma, resistance of prolactinoma to dopamine agonists was reported to be associated with high ESM1 expression. In GH3 and MMQ cells, silencing *ESM1* was reported to increase sensitivity to dopamine agonists and downregulated factors such as VEGFR2 and EGFR (23). However, this study had a small sample size and was based mainly on a single pair of cell lines; its mechanistic conclusions remain preliminary and require independent validation in larger clinical cohorts and additional *in vivo* models. Moreover, because no ESM1-targeted strategy has yet entered clinical trials, these findings should not be interpreted as evidence of imminent clinical translation.

### 3.4 Reprogramming of glycolysis and lipid/cholesterol metabolism

Preclinical studies suggest that ESM1 may participate in tumor cell energy metabolism through multi-level mechanisms, including glycolytic redirection, lipid metabolic reprogramming, and regulation of the autophagy–cholesterol network. However, these findings are largely based on a limited number of cell lines and animal models, and no ESM1-targeted metabolic strategy has yet entered clinical trials. They should therefore be regarded as preclinical and hypothesis-generating.

At the level of glycolysis regulation, in cervical cancer cells, HPV-driven ESM1 has been reported to upregulate the expression of HIF-1α and glycolytic enzymes by activating the Akt/mTOR pathway, which may promote aerobic glycolysis in cervical cancer cells to meet their bioenergetic and biosynthetic demands (43). In a hypoxic model of OC, ESM1 has been reported to act as a bridge to facilitate the interaction between PKM2 and the SUMOylation enzyme UBA2, inducing PKM2 SUMOylation and PKM2 dimer formation, which may enhance the Warburg effect and may promote fatty acid synthesis and vasculogenic mimicry (17). These mechanistic findings remain preclinical and require further *in vivo* and clinical validation. In addition, they are mainly derived from cervical and OC cell lines and xenograft models, and independent validation in additional models and clinical samples is still required.

At the level of lipid metabolic regulation, in OC, ESM1 has been reported to interact with ANGPTL4, which may promote the binding of ANGPTL4 to LPL and which may promote lipid metabolic reprogramming (52). In the regulation of cholesterol metabolism, ESM1 has been reported to regulate the KLF10/BECN1 axis and downregulate BECN1-mediated autophagy; the reduction in BECN1 may not only inhibit autophagy-mediated lipolysis but may also attenuate its ability to competitively bind HMGCR with HSPA5, thereby potentially reducing the ubiquitination and degradation of HMGCR, the rate-limiting enzyme in cholesterol synthesis, and consequently promoting cholesterol synthesis. In addition, elevated cholesterol levels have been reported to upregulate IGF2BP3, which may stabilize *ESM1* mRNA to form a positive feedback loop, thereby continuously driving lipid metabolic reprogramming (38). Although these metabolic mechanisms are supported by cell and animal experiments, independent validation in human tumors is still needed. Moreover, these findings are largely based on a limited number of cell lines and xenograft models, and no ESM1-targeted metabolic therapy has yet entered clinical trials.

In summary, the currently available preclinical evidence indicates that ESM1 may participate in tumor metabolic reprogramming by regulating glycolysis, lipid synthesis, and cholesterol metabolism. Its metabolic regulation under hypoxic conditions and the cholesterol positive feedback loop suggest that ESM1 may be one of the important regulators of tumor metabolic adaptation; however, the relevant mechanisms are mainly derived from cell and animal models and still require clinical validation. At present, all ESM1-targeted strategies remain preclinical, and no clinical trial has yet been initiated. Therefore, any clinical translation of these metabolic findings remains speculative and requires prospective validation.

### 3.5 ESM1-mediated immune remodeling and immune checkpoint therapy

ESM1 remodels the tumor immune microenvironment at multiple levels by regulating endothelial cell function, interfering with lymphocyte infiltration, and participating in oncogenic signaling. Its effects are cell source-dependent and context-specific. A growing body of evidence indicates that ESM1 can influence distinct immune cell populations, including myeloid cells and T lymphocytes, although the strength of evidence varies considerably across models. However, most of these findings are derived from a limited number of cell lines and preclinical models, and no ESM1-targeted immune strategy has yet entered clinical trials. They should therefore be regarded as preclinical and hypothesis-generating.

Regarding the impact of ESM1 on myeloid cells and the immune microenvironment, Qiu et al. (78) analyzed the association between endothelial cell subpopulation abundance and immune cell infiltration in BCa. Their results showed that patients with a higher abundance of the ESM1-high S1 endothelial cell subpopulation had worse clinical prognosis and increased infiltration of immunosuppressive cells. Further analysis revealed that the signature score of this ESM1-high endothelial cell subpopulation was positively correlated with the levels of immunosuppressive immune cell infiltration, including regulatory T cells and M2 macrophages. Qiu et al. (78) proposed that the ESM1-high endothelial cell subpopulation is associated with an immunosuppressive tumor microenvironment in BCa, and that targeting ESM1 in tumor-associated endothelial cells may represent a potential strategy for treating BCa and enhancing antitumor immunity. However, whether the relationship between this subpopulation and regulatory T cells (Tregs)/M2 macrophage infiltration is causal or merely concomitant, as well as the specific molecular mechanisms by which it regulates the immune microenvironment, remains to be further experimentally validated. Another multi-omics study of BCa speculated that ESM1 may suppress immune cell levels through the SPP1 signaling pathway. Its expression was negatively correlated with the infiltration levels of multiple immune cell types, including CD4+ T cells, CD8+ T cells, dendritic cells, and macrophages. In HCC and LUAD, SPP1 can mediate M2 polarization of macrophages and upregulate programmed death-ligand 1 (PD-L1), thereby attenuating the activation of CD4+ and CD8+ T cells. In addition, ESM1 forms a positive feedback loop with VEGF, and VEGF can inhibit T-cell development and function; therefore, ESM1 may also reduce T-cell infiltration by upregulating VEGF (79). Overall, ESM1 may contribute to the formation of an immunosuppressive tumor microenvironment by suppressing multiple antitumor immune cell populations, and may hold promise as a biomarker for predicting immune-related progression in BCa. However, these mechanisms remain largely speculative in BCa and require experimental validation. Moreover, these conclusions are based mainly on bioinformatics and correlation analyses rather than direct functional experiments.

Regarding CD8+ T-cell infiltration and cytotoxicity, studies have shown that ESM1 inhibits DNA damage repair (DDR) through the Akt1–MDM2–p53 axis, thereby reducing dsDNA production and subsequently suppressing the cGAS–STING pathway as well as downstream chemokine and interferon transcription, ultimately attenuating CD8+ T-cell infiltration. *In vivo*, *ESM1* knockout enhanced CD8+ T-cell infiltration and inhibited tumor growth, and patient samples further demonstrated a negative correlation between ESM1 and CD8 expression (37). However, the effects of ESM1 on other immune cell populations, including natural killer (NK) cells, Tregs, B cells, and dendritic cells, remain unclear and warrant further investigation. In OC, lactate can upregulate ESM1, which in turn enhances SCD1 activity and activates the Wnt/β-catenin pathway, ultimately attenuating the antitumor CD8+ T-cell response. Meanwhile, the ESM1–SCD1 axis promotes resistance to platinum-based drugs such as cisplatin by upregulating P-gp (36). These mechanistic findings remain preclinical and are based on a limited number of cell lines and animal models; independent validation in human samples and prospective clinical cohorts is still required.

Regarding CD4+ T-cell immune evasion, Li et al. (12) found that in HCC, BCL6 upregulates *ESM1* expression. As a downstream immunosuppressive molecule, ESM1 inhibits CD4+ T cell-mediated tumor cytotoxicity, thereby promoting immune evasion. The underlying mechanism may involve ESM1 binding to lymphocyte function-associated antigen 1 (LFA-1) and interfering with intercellular adhesion molecule 1 (ICAM-1)/LFA-1 signaling, consequently suppressing lymphocyte recruitment and activation. The study also found that BCL6 can inhibit CD8+ T cells; however, whether ESM1 directly mediates this effect remains unclear. These conclusions are based mainly on HCC cell lines and require validation in additional models and clinical samples.

Regarding the relationship between oncogenic signaling and immune remodeling, in melanoma, high ESM1 expression is associated with activation of the Wnt/β-catenin and NOTCH signaling pathways, accompanied by increased numbers of multiple immune-related cell populations and upregulated expression of immune-related genes in the tumor microenvironment (67). This finding suggests that ESM1 may participate in the crosstalk between intrinsic oncogenic signaling in tumor cells and immune remodeling of the microenvironment. However, the study did not distinguish specific immune cell subsets, but merely suggested an association between ESM1 and increased immune cell infiltration. These conclusions are mainly based on preclinical models and retrospective data, and prospective clinical validation is still lacking.

Regarding immune checkpoint therapy, given that the ESM1-high vascular niche is characterized by reduced CD8+ T-cell infiltration and enrichment of M2 macrophages, myeloid-derived suppressor cells (MDSCs), and Tregs, it would theoretically be expected to adversely affect the response to immune checkpoint blockade (ICB). In the IMvigor210 anti-PD-L1 cohort, the objective response rate did not differ significantly between patients with high versus low S1 EC abundance (P = 0.28) (78). This suggests that ESM1 alone is insufficient to predict anti-PD-L1 response. Actual benefit also depends on a multi-factorial immune context, including PD-L1 expression, tumor mutational burden, and prior treatments. Similarly, after stratification by *ESM1* expression, no statistically significant differences in response to anti-programmed cell death protein 1 (PD-1) or anti-cytotoxic T-lymphocyte-associated protein 4 (CTLA-4) therapy were observed between patients in the high- and low-expression groups, further suggesting that ESM1 is more likely a marker of an immunosuppressive microenvironment than an independent predictor of ICB response (79). Notably, differences exist across tumor types: in an eight-gene immune prognostic model incorporating ESM1 for diffuse large B-cell lymphoma (DLBCL), the high-risk group exhibited upregulation of PD-L1, B- and T-lymphocyte attenuator (BTLA), and sialic acid-binding immunoglobulin-like lectin 7 (SIGLEC7), and was predicted, based on its molecular features, to derive greater benefit from ICB (80), suggesting that the predictive value of ESM1 is context-dependent. This indicates that the predictive value of ESM1 is not universally applicable across all tumor types and should therefore be interpreted with caution and assessed within specific contexts. Several factors may explain this inconsistency. First, the cellular source of ESM1 differs across tumors: in BCa, ESM1 is predominantly expressed by immunosuppressive endothelial subpopulations (S1 endothelial cells [S1 ECs]) and mainly reflects a vascular immune barrier (78), whereas in DLBCL it may be co-expressed with immune checkpoint molecules such as PD-L1, BTLA, and SIGLEC7 and thus identify a more immunogenic subgroup (80). Second, the immune microenvironment composition differs; ESM1-high niches in BCa are enriched with M2 macrophages and MDSCs, which may not be reversed by ICB monotherapy (78, 79),. Third, treatment context matters: the IMvigor210 cohort received anti-PD-L1 monotherapy (78), whereas combining anti-angiogenic therapy with ICB reduces ESM1+ endothelial cells and increases CD8+ T-cell infiltration (81). Fourth, ESM1 glycosylation status and differences in detection methods or cut-off values across studies may also contribute. Therefore, ESM1 should be regarded as a component of the immunosuppressive microenvironment rather than a standalone ICB predictor. To date, the most direct evidence comes from combination treatment models: in high-grade serous OC, anlotinib plus anti-PD-L1 therapy increased the disease control rate from 71.43% to 100%, accompanied by a reduction in ESM1+ endothelial cells, increased CD8+ T-cell infiltration, reversal of T-cell exhaustion, and M1 polarization of macrophages (81). These preclinical findings suggest that targeting the ESM1-related axis may remodel the vascular niche and reverse the immunosuppressive microenvironment, thereby enhancing the efficacy of immune checkpoint blockade. However, no clinical trial has yet tested this combination, and prospective clinical validation is still lacking. These explanations are largely speculative and based on preclinical or retrospective evidence; they require further experimental and clinical validation.

Preclinical evidence suggests that ESM1 may influence the tumor immune microenvironment through endothelial-cell remodeling, tumor-cell immune evasion, and intercellular signaling. The diversity of its functions raises the possibility that targeting ESM1 may simultaneously influence angiogenesis and immunosuppression, which could offer potential for synergistic therapies, particularly in combination with immune checkpoint blockade. However, most current evidence remains preclinical or retrospective, and no ESM1-targeted strategy has yet entered clinical trials. Prospective clinical validation is still lacking. These findings highlight the context-dependent nature of ESM1-mediated immune remodeling and the need for future studies to distinguish cellular sources and validate these mechanisms prospectively. (**Figure 2**)

**Figure 2:**
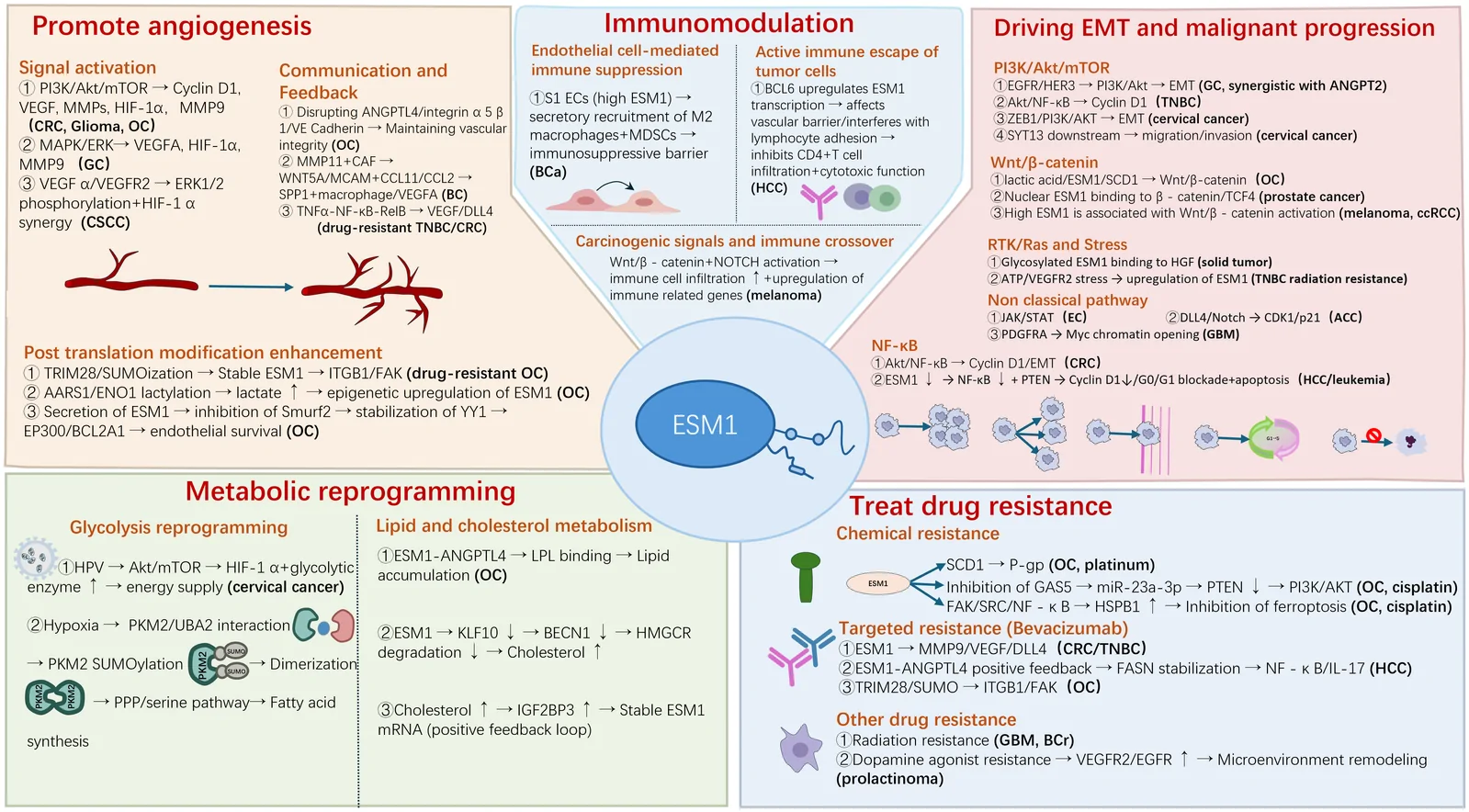
Multidimensional regulatory network of ESM1 in tumorigenesis. The diagram summarizes five major functional modules: (1) promotion of angiogenesis; (2) cell–cell communication and positive feedback loops; (3) remodeling of the tumor immune microenvironment; (4) EMT and malignant progression; and (5) therapeutic resistance and metabolic reprogramming. Dashed arrows indicate crosstalk among modules. See the main text for detailed mechanisms.

## 4 Limited evidence supports a context-dependent antitumor role of ESM1

### 4.1 Context-dependent tumor-suppressive effects reported in prostate cancer, GC, and CRC

Current evidence indicates that ESM1 does not act exclusively as a tumor-promoting factor in all contexts. In a limited number of specific tumor types and under particular experimental conditions, a tumor-suppressive role of ESM1 has been reported. However, it should be emphasized that the evidence supporting a tumor-suppressive function of ESM1 is currently restricted to the following three studies, all of which are limited to specific cell lines and experimental systems. The strength of this evidence is far weaker than that of the substantial literature supporting its tumor-promoting functions and cannot be regarded as equally established. Moreover, all of these findings remain preclinical, and no ESM1-targeted therapy has yet entered clinical trials. The following observations should therefore be considered context-dependent and preliminary rather than established evidence for ESM1 as a canonical tumor suppressor.

In prostate cancer, *ESM1* knockdown has been reported to downregulate the cell cycle regulator p21 and upregulate Cyclin D1, thereby accelerating cell cycle progression. Concurrently, *ESM1* deficiency has been reported to lead to decreased tissue inhibitor of metalloproteinase-1 (TIMP‐1) and increased matrix metalloproteinase-9 (MMP‐9) expression, disrupting the MMP‐9/TIMP‐1 balance, remodeling the tumor microenvironment, and promoting invasion. Importantly, recombinant human TIMP‐1 protein significantly reverses the *ESM1*‐knockdown‐induced enhancement of migration and invasion, whereas anti‐TIMP‐1 antibody blocks this reversal, suggesting that the TIMP‐1/MMP‐9 axis is a key downstream pathway through which ESM1 regulates prostate cancer progression. *In vivo* studies are consistent with these findings: PC3 and DU145 cells with *ESM1* knockdown exhibit significantly increased tumor volume and weight in a subcutaneous xenograft model, along with elevated Ki‐67 proliferation indices. In a tail‐vein injection lung metastasis model, the *ESM1*‐knockdown group displayed a marked increase in the number of pulmonary metastatic nodules, and histological examination revealed a higher proportion of Ki‐67‐positive cells within the metastatic foci (82). These findings are contrary to the previously reported tumor-promoting effects of ESM1 in GC, CRC, and HCC. To explain this discrepancy, the authors proposed a speculative glycosylation balance hypothesis, drawing on the study by Yassine et al. Yassine et al. (83) found that the glycosylation level of mouse ESM1 is much lower than that of the human protein, and that it exists in serum in both glycosylated and non-glycosylated forms. Its glycosylation pattern is determined by the N-terminal domain encoded by exons 1/2, which differs by 12% between human and mouse. Functionally, in specific experimental models, fully glycosylated human *ESM1* has been reported to promote tumor growth through mitogenic effects synergistic with FGF-2 and HGF/SF, as well as through chemotactic effects on endothelial cells. In contrast, in specific experimental models, the poorly glycosylated ESM1 polypeptide has been reported to bind LFA-1 and inhibit LFA-1/ICAM-1 interaction, thereby delaying tumor growth in an HT-29 xenograft model; this effect is dependent on CD122+ leukocytes (NK cells in SCID mice). Accordingly, Yassine et al. speculated that the balance between glycosylated and non-glycosylated ESM1 may help determine whether ESM1 promotes or inhibits tumor growth. However, this remains a hypothesis proposed to explain the experimental discrepancies. Although Yassine et al. directly compared non-glycosylated ESM1 mutants with glycosylated forms, the overall hypothesis that a glycosylation/non-glycosylation balance determines the functional output of ESM1 has not been demonstrated by direct experimental manipulation, nor has it been directly validated in tumor tissues or in the tumor microenvironment. No direct evidence is currently available as to whether the balance between glycosylated and non-glycosylated ESM1 is shifted within tumor tissues or whether the tumor microenvironment regulates the glycosylation status of ESM1. It should be noted that, among the three tumor-suppressive studies, only the prostate cancer study included *in vivo* validation, and even this evidence remains preclinical.

In GC, *ESM1* expression levels are positively correlated with the degree of tumor differentiation. Overexpression of *ESM1* suppresses cell migration, promotes differentiation, and induces apoptosis, whereas *ESM1* knockdown produces the opposite phenotype, suggesting that ESM1 attenuates the malignant phenotype of tumor cells in this model, involving the regulation of p53 and caspase-3. Zhang et al. demonstrated that *ESM1* overexpression causes SGC7901 cells to adopt a round, well‐differentiated morphology, accompanied by a significant reduction in the activities of the differentiation marker enzymes alkaline phosphatase (AKP) and lactate dehydrogenase (LDH). In contrast, *ESM1* knockdown results in a polygonal or irregular, poorly differentiated morphology with elevated AKP and LDH activities. Hoechst 33342 staining showed a marked increase in apoptotic cells in the *ESM1*-overexpression group, whereas fewer apoptotic cells were observed in the knockdown group. Western blot analysis confirmed that *ESM1* overexpression upregulates p53 and caspase‐3 protein levels, whereas knockdown downregulates both. Furthermore, enzyme-linked immunosorbent assay (ELISA) measurements demonstrated that the concentration of secreted ESM1 in the culture supernatant was significantly elevated in the overexpression group and reduced in the knockdown group, confirming that ESM1 acts in an autocrine/paracrine manner (84). The same group’s previous work further demonstrated that melatonin significantly upregulated *ESM1* expression, decreased the activities of dedifferentiation-associated enzymes such as AKP and LDH, and promoted a more mature, differentiated phenotype, thereby exerting an antitumor effect. However, this study was limited to a single cell line, SGC7901, and no *in vivo* validation was performed (84). Therefore, these findings should be regarded as preliminary and context-dependent, and independent validation in additional cell lines, animal models, and clinical samples is still required.

In CRC, butyrate relieves the inhibition of *ESM1* by suppressing MAPK/ERK2 signaling pathway phosphorylation, thereby upregulating *ESM1* expression and subsequently inhibiting CRC proliferation, migration, and clonogenicity. Mechanistically, butyrate dose‐dependently inhibits the phosphorylation of ERK1/2 and p38. Treatment with the MEK/ERK inhibitor PD98059 further enhances butyrate‐induced *ESM1* upregulation, whereas the MAPK activator PMA reverses this effect. Knockdown of ERK2 in RKO cells significantly increases both *ESM1* mRNA and protein levels, confirming that the ERK2/MAPK pathway negatively regulates *ESM1* expression. Functionally, *ESM1* overexpression alone suppresses RKO cell proliferation, migration, and soft‐agar colony formation, and induces cell cycle arrest at the S phase, while *ESM1* knockdown promotes these malignant behaviors. More importantly, in *ESM1*‐knockdown cells, the inhibitory effects of butyrate on proliferation, migration, and colony formation are substantially attenuated, indicating that ESM1 is a critical downstream effector of butyrate’s anticancer activity. In addition, Annexin V‐FITC/PI double‐staining flow cytometry and Hoechst staining confirmed that butyrate induces apoptosis in RKO cells, but this apoptotic effect is weakened in *ESM1‐*knockdown cells (41). However, this evidence was obtained mainly from the RKO cell line and *in vitro* functional experiments, and *in vivo* validation was not provided. Therefore, these findings support a context-dependent antitumor effect of ESM1 in this model but do not establish ESM1 as a universal tumor suppressor in CRC. Moreover, no clinical data are available to support the tumor-suppressive role of ESM1 in CRC, and the therapeutic implications of these findings remain speculative (**Figure 3**).

**Figure 3:**
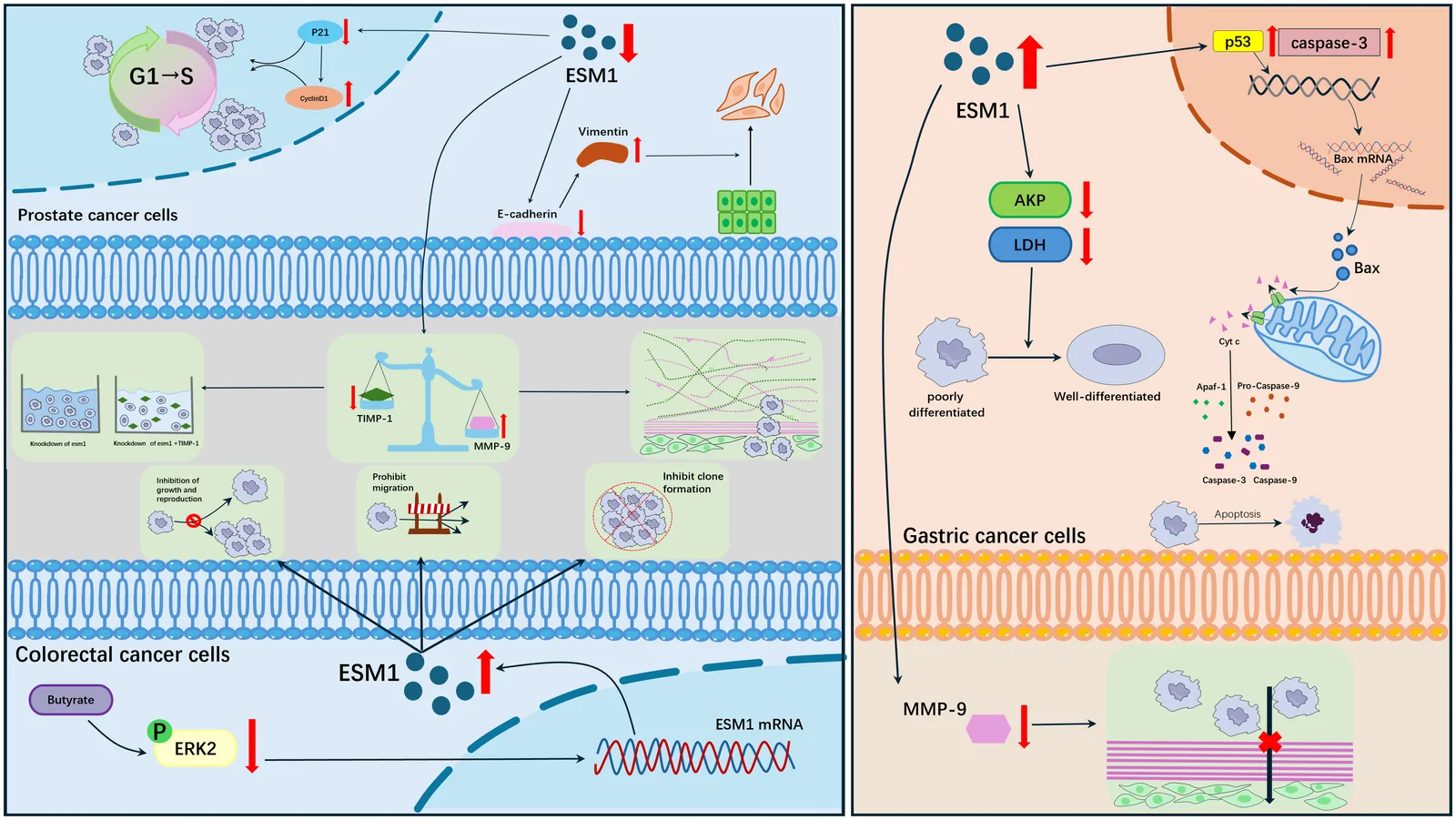
In prostate cancer, *ESM1* knockdown promotes proliferation, migration, invasion, and EMT through dysregulation of p21/cyclin D1 and TIMP-1/MMP-9. In GC, ESM1 promotes apoptosis, inhibits migration/invasion, and induces differentiation via p53/caspase-3, MMP-9, and differentiation markers. In CRC, butyrate upregulates *ESM1* by inhibiting ERK2/MAPK, thereby suppressing proliferation, migration, and colony formation. See the main text for detailed mechanisms.

### 4.2 Common patterns, key gaps, and cross-cancer synthesis: key microenvironmental determinants

Common patterns. The three tumor-suppressive studies were restricted to prostate cancer, GC, and CRC. In GC and CRC, ESM1 protein expression in tumor tissues has been reported to be lower than in normal tissues and positively correlated with differentiation; however, the prostate cancer study did not analyze ESM1 protein expression in tumor tissues. All three studies were based on a small number of specific cell lines, and only the prostate cancer study included *in vivo* validation. The downstream pathways overlap minimally: prostate cancer involves p21/cyclin D1 and the TIMP-1/MMP-9 axis; GC involves p53/caspase-3 and MMP-9; and CRC involves the MAPK/ERK2–*ESM1* axis. The only notable intersection is that ESM1 negatively regulates MMP-9 in both prostate cancer and GC, which has not been demonstrated in CRC.

Key gaps. No shared core mechanism has been established, and the tumor-suppressive role of ESM1 cannot yet be generalized across cancers. *In vivo* evidence is limited to prostate cancer, and independent validation in additional models and tumor types is lacking. Although glycosylation status has been proposed to influence the functional direction of ESM1, the three tumor-suppressive studies did not directly manipulate ESM1 glycosylation, and the ratio of glycosylated to non-glycosylated ESM1 in human tumors and its relationship with tumor phenotype remain unknown. Although Yassine et al. directly compared non-glycosylated ESM1 mutants with glycosylated forms, the overall hypothesis that a glycosylation/non-glycosylation balance determines functional output remains speculative and has not been demonstrated by direct experimental manipulation, nor directly validated in tumor tissues or the tumor microenvironment. Whether the tumor-suppressive effect depends on cellular source, disease stage, or other microenvironmental factors also remains untested. Clinical validation is still lacking.

Cross-cancer synthesis and key microenvironmental determinants. The contradictory phenotypes of ESM1 across cancer types cannot be explained by tumor type alone. Instead, the functional output of ESM1 appears to be determined by specific microenvironmental variables that have not yet been clearly defined or systematically compared. These candidate determinants include cellular source (endothelial vs. tumor vs. stromal), glycosylation status, immune context, metabolic and hypoxic signals, and experimental model and disease stage. None of these variables has been systematically manipulated across tumor types, and no cross-cancer study has directly compared them. Therefore, the switch between pro- and anti-tumor functions of ESM1 remains poorly defined, and the tumor-suppressive activity of ESM1 should be interpreted as a highly context-dependent preliminary observation. Its universality, determinants, and relationship with glycosylation status remain to be validated by more systematic experiments.

## 5 Clinical translation and therapeutic potential

### 5.1 Diagnostic value: from early screening to benign-malignant differentiation

Early detection and accurate staging of tumors are critical for improving patient prognosis, and an ideal biomarker should provide useful information across multiple stages of disease initiation and progression. ESM1 is aberrantly expressed in various tumor types and is closely associated with the degree of malignancy, suggesting its potential as a candidate biomarker throughout the entire process of tumor development. However, ESM1 is not only secreted by activated vascular endothelial cells but can also be expressed by some tumor cells and stromal cells (32). Circulating ESM1 may be affected by inflammation, endothelial activation, and tissue injury. Currently, the diagnostic evidence for ESM1 is mainly based on bioinformatics associations and retrospective clinical studies, and prospective, multicenter independent validation is still lacking; considerable differences in detection specimens, antibodies, and cut-off values across studies also limit the comparability of results (**Table 1**).

**Table 1:** ESM1 expression, mechanisms, and clinical significance in cancer.

Cancer type	Sample type	Body fluid testing	ESM1	Function (keymechanism)	Clinical significance	References
GC	Cell lines, tissues	Serum	↑	Carcinogenic (EGFR/HER3-Akt-ANGPT2)	Poor prognosis; adjuvant chemotherapy predictor	(57, 85)
	Cell lines, tissues	—	↓	Anticancer (p53/caspase-3, MMP-9 melatonin-induced differentiation)	Inhibits migration; promotes differentiation/apoptosis; differentiation marker	(39, 84)
CRC	Cell lines	Serum, urine, saliva (proposed)	↑	Carcinogenic (PI3K/Akt/mTOR; Akt/NF‐κB; MMP9/VEGF/DLL4)	Poor prognosis; recurrence risk; anti-ESM1+bevacizumab	(27, 47, 54, 62, 86)
	Cell lines	—	↑	Anticancer (Inhibit MAPK/ERK2)	Potential therapeutic target	(41)
HCC	Tissues, Cell lines	Serum	↑	Carcinogenic (NF‐κB/PTEN/Cyclin D1)	Superior to AFP;MTM-HCC marker; early recurrence	(63, 87–90)
EC	Tissues, Cell lines	—	↑	Carcinogenic (ESM1-JAK)	Worse N stage/pTNM; shorter OS/PFI/DSS	(70, 91, 92)
OC	Tissues, Cell lines	Serum	↑	Carcinogenic (ANGPTL4;SCD1/Wnt; PI3K/AKT)	Poor prognosis; anlotinib+anti-PD-L1 DCR 100%	(21, 36, 52, 81)
Cervical	Tissues, Cell lines	—	↑	Carcinogenic (ZEB1/PI3K/AKT; Akt/mTOR/HIF-1α)	Independent prognostic factor; aloe-emodin target	(43, 59)
BC	Tissues, Cell lines	Serum, Plasma	↑	Carcinogenic (Akt/NF‐κB; ATP/VEGFR2)	Recurrence>60%; poor prognosis; radiation resistance	(29, 42, 58, 77, 93)
BCa	Tissues, Cell lines	Serum, Urine	↑	Carcinogenic (WNT5A/MCAM; CCL2/CCL11-VEGFA; S1 ECs)	Muscle invasion; poor prognosis; urine diagnosis; anti-VEGF prediction	(20, 53, 78, 94, 95)
Prostate cancer	Tissues, Cell lines	Serum	↑	Carcinogenic (Nuclear ESM1-β-catenin-Wnt)	Postoperative recurrence predictor	(66, 96, 97)
	Tissues, animal models	—	↓	Anticancer (TIMP-1/MMP-9 imbalance; p21/Cyclin D1)	Low expression may promote invasion	(82)
ccRCC	Tissues, Cell lines, animal models, databases	Serum	↑	Carcinogenic (Akt/mTOR; Ras; Wnt/β-catenin)	Poor prognosis; anti-vascular response	(19, 51, 68, 98)
ACC	Tissues, Cell lines	—	↑	Carcinogenic (DLL4-Notch→CDK1/p21)	Benign vs malignant differentiation	(72, 99)
Glioma	Tissues, Cell lines	Serum	↑	Carcinogenic (PDGFRA/Myc; radiation resistance)	Poor prognosis; postoperative monitoring	(10, 24, 73, 100)

“↑” indicates upregulated/high ESM1 expression, and “↓” indicates downregulated/low ESM1 expression.

In terms of early diagnosis and screening, the sources of evidence are highly heterogeneous. ESM1 can be detected in precancerous lesions and early-stage tumors. As cervical squamous intraepithelial lesions progress to invasive carcinoma, ESM1 exhibits a staged “low–high–plateau” distribution pattern, suggesting that its high expression may be associated with the development of cervical squamous cell tumors (101). However, this study was a retrospective observational immunohistochemical study of tissue and did not distinguish the source of positively stained cells; therefore, it reflects a local expression pattern rather than a circulating biomarker suitable for population screening. In CRC, a 7-gene signature comprising *ESM1* can distinguish tumors from normal tissues with an accuracy exceeding 98% (102). However, this model was constructed using machine learning based on TCGA transcriptomic data and thus constitutes bioinformatics-based predictive evidence; the >98% accuracy represents the performance of a multi-gene combination, from which the independent contribution of *ESM1* cannot be isolated, and the authors also stated that this tool still requires clinical validation. In GC and EC, ESM1 exhibited high diagnostic value as early as stage I (area under the curve [AUC] > 0.79, exact AUC value and 95% CI were not reported) (92). However, this study had a small sample size, with 36 cases each for GC and EC, and mainly used quantitative real-time PCR (RT-qPCR) and Western blot. Its diagnostic performance evaluation was not based on a standardized clinical immunoassay platform, and the cut-off values and detection platform were not reproduced in independent cohorts; therefore, its AUC should be regarded as a preliminary estimate. Machine learning-based bioinformatics analysis also predicted *ESM1* in AML (103), HCC (104), and early-stage BCa (105). However, these findings all constitute bioinformatics-based predictive evidence: the AML study itself noted the lack of external data validation; the liver cancer study was purely computational prediction; and in the BCa study, ESM1 served only as one component of a 14-protein combined panel, with its standalone diagnostic performance not reported. Therefore, these results should not be equated with clinically validated conclusions. Moreover, because no ESM1-targeted diagnostic or therapeutic strategy has yet entered clinical trials, any clinical translation remains speculative.

In terms of auxiliary diagnosis, ESM1 has been reported to show relatively high diagnostic performance in CRC and GC [CRC: accuracy = 0.94, sensitivity = 99%, specificity = 73%; 95% CI not reported (86); GC: accuracy = 0.946, sensitivity = 98%, specificity = 80%; 95% CI not reported (106)]. However, the two studies differed in their units of measurement and in the way cut-off values were reported, and a standardized assay system is lacking, making the values difficult to compare directly. Regarding the diagnosis of liver cancer, multiple studies have suggested that ESM1 exhibits favorable diagnostic performance. In a representative study ROC curve analysis of 40 patients with HCC and a control group (including patients with hepatitis, cirrhosis, and healthy individuals) showed that the accuracy of ESM1 was higher than that of the conventional marker alpha-fetoprotein (AFP). However, IHC in this study showed that ESM1 was expressed in the endothelium of HCC tissues but not in normal liver tissues. In addition, *in vitro* experiments confirmed that ESM1 is involved in the proliferation, cell cycle progression, migration, and invasion of HCC cells; however, the latter constitutes evidence from *in vitro* cell experiments and cannot be extrapolated to clinical diagnostic performance. The study ultimately proposed that ESM1 alone or ESM1 combined with AFP may be a useful biomarker for the diagnosis of HCC (63, 87). However, these conclusions were derived from a single-center, small-sample cohort, without standard blinded validation or testing in multicenter, real-world screening populations. Therefore, these conclusions can currently be regarded only as hypothesis-generating results; the value of ESM1 more likely lies in its combination with indicators such as AFP, but this still requires validation. In particular, it should be emphasized that the diagnostic evidence for ESM1 is not consistent across cancer types. In BCa, for example, a non-randomized, prospective, case-control study (154 patients with primary BCa vs. 52 healthy controls) showed that serum ESM1 for diagnosing BCa had an AUC of only 0.609 (95% CI 0.524–0.694), with a sensitivity of 59.1% and a specificity of 57.7%, which is far below the approximately 0.94 magnitude reported for gastrointestinal tumors; however, it should be noted that the detection platforms, sample types, and populations differed between the two. The same study also found that serum ESM1 levels were positively correlated with adverse pathological features such as high grade and muscle invasion (94). Therefore, the value of ESM1 as a “diagnostic” marker for BCa is limited, but it may serve as a marker associated with the “degree of invasion/malignant phenotype”; these two aspects should be evaluated separately. Another study involving 50 patients with BCa showed that urinary ESM1 had a sensitivity of 62% and a specificity of 71%; urinary ESM1 was also significantly elevated in patients with urinary tract infection and showed no statistically significant difference from that in the BCa group (95). This suggests that infection and inflammation may elevate ESM1, representing confounding factors that should be considered when interpreting urinary ESM1. The evidence in hematologic malignancies is similarly weak: plasma ESM1 distinguished newly diagnosed multiple myeloma from monoclonal gammopathy of undetermined significance (MGUS) with an AUC = 0.66 (cut-off >220 pg/mL; 95% CI not reported) (107). At the level of urinary cell-free RNA, the AUC = 0.704; 95% CI not reported, and the diagnostic marker ultimately selected was ubiquitin-conjugating enzyme E2 C (UBE2C) rather than *ESM1* (108). In summary, ESM1 exhibits promising diagnostic signals in some gastrointestinal tumors, whereas its performance is limited in other cancer types such as BCa and myeloma. The currently available evidence is insufficient to support ESM1 as a cross-cancer diagnostic biomarker, and its diagnostic value in different cancer types needs to be validated separately. At present, all ESM1-targeted strategies remain preclinical, and no clinical trial has yet been initiated. In addition, high expression of ESM1 has also been observed in solid tumors such as lung cancer and BCa, as well as in pituitary adenomas (PA) and hematological malignancies (94, 96, 100, 107–112). However, most of these are tissue expression or small-sample correlation studies, which suggest the prevalence of ESM1 expression but do not equate to diagnostic validity.

In the differentiation between benign and malignant lesions and the assessment of invasiveness, ESM1 expression levels have shown potential value in some tumor-related samples. In NSCLC, ESM1 levels in malignant pleural effusion were significantly higher than those in benign effusion; in that study, the sensitivity and specificity of ESM1 for diagnosing malignant pleural effusion were 81.4% and 84.0%, respectively (cut-off 19.58 ng/mL), and high ESM1 levels in malignant pleural effusion (MPE) were independently associated with shorter overall survival (OS) (113). This study had a retrospective design and did not perform a head-to-head comparison with current differential diagnostic methods such as cytology; elevated ESM1 in pleural fluid may also partly reflect pleural inflammation and vascular leakage. In ACC, ESM1 showed positive expression in both ACC and adrenocortical adenoma, with a positivity rate of 100% in both; however, the percentage of ESM1-positive cells was significantly higher in carcinoma tissues than in adenoma and normal adrenal cortex (99). However, this study had a very small sample size (7 ACC cases, 6 ACA cases, and 13 normal cases), and the positivity rate of ESM1 in adenomas was also 100%, with 30.7% expression in normal adrenal cortex, suggesting that ESM1 positivity/negativity alone has limited discriminatory power, with only statistically significant differences in expression intensity or the proportion of positive cells. In addition, as a vascular endothelial-secreted protein, ESM1 levels may also be elevated in conditions such as non-neoplastic inflammation, tissue injury, and angiogenesis. The above studies did not systematically evaluate these confounding factors, which limits the potential of ESM1 for clinical translation as an independent differential diagnostic marker.

In terms of invasiveness assessment, ESM1 levels are associated with the degree of tumor malignancy. In PAs, ESM1 expression was strongly correlated with the Knosp invasion grade, whereas conventional vascular markers such as CD34 and CD105 showed no such association. This study compared the associations of ESM1, CD34, and CD105 with the Knosp grade: ESM1 (predominantly tumor cell staining; Spearman r = 0.616) was superior to CD34/CD105-based microvessel density (MVD) (r = 0.142/0.166), suggesting that overexpression of tumor cell-derived ESM1 may better reflect the invasiveness of PAs than MVD, and it was speculated that angiogenesis may not be the main driver of ESM1-mediated invasion (114). Another study that included only null cell adenomas reached partially different conclusions: vascular ESM1 expression was associated with invasion, whereas ESM1 expression in adenoma tissue was only associated with tumor size (115). The discrepancy between the conclusions of the two studies—namely, whether tumor-derived or endothelial-derived ESM1 better reflects invasiveness—remains unresolved and may be attributable to the marked heterogeneity of PAs and differences in detection methods. This suggests that caution should be exercised when extrapolating findings from PAs to other cancer types. In BCa, serum ESM1 levels in patients were higher than those in healthy controls and were associated with adverse pathological features, including stage, grade, muscle-invasive status, and tumor volume (94). These findings suggest that ESM1 may serve as a molecular marker of invasive behavior; however, all currently available evidence is correlative, and the causal relationship between ESM1 and invasion still needs to be verified through functional experiments. Furthermore, no ESM1-targeted therapy has entered clinical trials, so these findings should not be interpreted as evidence of imminent clinical translation.

In terms of identifying high-risk subtypes and monitoring metastasis, ESM1 shows potential for molecular subtyping. In HCC, ESM1 has been identified as a specific immunohistochemical marker for the macrotrabecular-massive hepatocellular carcinoma (MTM-HCC) subtype, which is associated with a high risk of recurrence, with both sensitivity and specificity exceeding 90% (88). However, this study detected ESM1 expression in stromal endothelial cells rather than in tumor cells, and the evidence was derived from two independent HCC biopsy series (67 and 132 cases), constituting a retrospective clinical study; therefore, its applicability is mainly limited to the pathological diagnosis of HCC subtypes. In a mouse model of BC lung metastasis, plasma ESM1 levels were significantly elevated at the early stage of metastasis, and the study interpreted this as reflecting pulmonary endothelial glycocalyx disruption/endothelial activation, suggesting that ESM1 may serve as a candidate marker of early metastasis-associated endothelial response (116), however, this evidence is derived from animal models and cannot be directly extrapolated to clinical metastasis monitoring indicators. In melanoma, a machine learning model based on TCGA transcriptomic data listed *ESM1* as one of the candidate markers for distinguishing metastatic from primary tumors (117). However, this model is a 17-gene signature, with an AUROC of 0.95 and an accuracy of 89.47% on an independent validation dataset, and the individual contribution of *ESM1* was not reported; this conclusion constitutes a bioinformatics-based prediction and still requires experimental validation.

In terms of liquid biopsy applications, ESM1 can be detected in body fluids such as serum and urine, suggesting its potential value for non-invasive detection. Urinary ESM1 levels were significantly elevated in patients with BCa and were higher than those in paired serum samples. The authors speculated that this may be related to the direct shedding of urothelial cells into urine, that is, part of urinary ESM1 may originate from bladder epithelial cells rather than solely from endothelium-derived molecules transported through the bloodstream. This suggests that the cellular sources of ESM1 may differ among body fluids and should be interpreted separately (95, 108). A study based on integrated multi-omics data prediction found that ESM1 can be detected in the blood, urine, and saliva of patients with CRC.

In summary, ESM1 shows some potential in tumor diagnosis and the assessment of malignant progression; however, the currently available evidence consists mainly of bioinformatics predictions and retrospective, single-center, small-sample studies, with inconsistent detection methods and cut-off values and marked differences in diagnostic performance across cancer types. The sources of circulating and body fluid ESM1 remain unclear, urinary tract infection and inflammation can directly elevate ESM1, and prospective head-to-head comparisons with existing markers such as AFP, carcinoembryonic antigen (CEA), cancer antigen 125 (CA-125), fluorescence *in situ* hybridization (FISH), and cytology are also lacking. Future studies should establish standardized assays, conduct prospective multicenter validation, include inflammatory controls, clarify cellular sources, and evaluate the value of ESM1 in combination with existing markers. At present, however, all ESM1-targeted strategies remain preclinical, and no clinical trial has yet been initiated; therefore, any clinical translation remains speculative and requires rigorous prospective validation.

### 5.2 Prognostic value: risk stratification and survival prediction

ESM1 is highly expressed in multiple digestive system tumors and is associated with survival, recurrence, and aggressive clinicopathological features, suggesting that it may serve as a candidate prognostic biomarker. From an evidence-based medicine perspective, a meta-analysis including 15 studies and 1,464 patients showed that high ESM1 expression was significantly associated with poorer OS (hazard ratio [HR] = 2.48, 95% confidence interval [CI] 2.12–2.90), P<0.001), with the strongest associations observed in gastrointestinal tumors (HR = 2.27, 95% CI 1.77-2.91, P<0.001) and HCC (HR = 2.61, 95% CI 1.96-3.48, P<0.001) (118). However, this meta-analysis may be subject to publication bias, inadequate adjustment for stage and treatment, and inconsistent detection methods across studies; therefore, this HR estimate should be interpreted with caution. In addition, most other available evidence also consists predominantly of retrospective, single-center studies, and some HRs were indirectly estimated from survival curves. Therefore, the comparability and robustness of the results are limited, and further validation is still required. Moreover, because no ESM1-targeted therapy has yet entered clinical trials, the prognostic value of ESM1 remains a research question rather than a clinically actionable tool.

In digestive system tumors, the evidence for the prognostic value of ESM1 is relatively abundant. In GC, high serum and tissue ESM1 expression is associated with aggressive features such as poor differentiation, lymph node metastasis, and advanced tumor-node-metastasis (TNM) stage; an IHC study involving 159 patients showed that ESM1 was an independent prognostic factor for OS (119, 120), however, most of these are retrospective cohort studies, and the independent prognostic value of ESM1 still requires validation under standardized criteria. In patients with stage II/III GC receiving adjuvant S-1 chemotherapy, high *ESM1* mRNA expression was independently associated with poorer OS (HR = 2.291, 95% CI not reported, P = 0.007) (121), however, this population has a specific treatment background and cannot be directly extrapolated to all GC patients. In CRC, both tissue and serum ESM1 levels were associated with deeper tumor invasion, lymph node metastasis, distant metastasis, and poorer differentiation; some studies reported that serum ESM1 independently predicted an increased risk of death (HR = 4.09, 95% CI 2.27–10.88, P < 0.001) (122–124). In addition, several prognostic models incorporating *ESM1* (such as an 8-gene immune-related model and a 4-gene immune-metabolic combined model) showed moderate predictive performance in TCGA and external cohorts (3–5-year AUC of approximately 0.70–0.79; exact AUC values and 95% CI were not reported), however, most of these models were constructed based on bioinformatics, and the mechanistic inferences remain descriptive, with functional experiments and prospective validation still lacking (125, 126). Importantly, because *ESM1* is only one component of these multi-gene signatures, its independent predictive contribution cannot be separated from that of the other genes, and these models should not be interpreted as evidence that *ESM1* alone has independent prognostic value. In HCC, ESM1 microvessel density is associated with microscopic venous invasion and shorter OS after curative hepatectomy; multivariate analysis confirmed endocan-MVD as an independent prognostic marker for OS (HR = 3.31, 95% CI 2.10–4.35, P < 0.01) (90). In addition, *ESM1* is a component of a multigene angiogenesis-related signature (ANGPT2, DLL4, NETO2, *ESM1*, NR4A1) that can identify rapidly growing HCC (training set AUC = 0.961); however, this signature was derived from a single-center cohort, and its generalizability remains to be validated (89, 127). As with other multi-gene signatures, the independent predictive value of *ESM1* cannot be isolated from the model, and the observed performance reflects the combined effect of all genes rather than *ESM1* alone. In EC, high ESM1 expression is associated with poorer N stage and pathological tumor-node-metastasis (pTNM) staging and predicts shorter OS, progression-free interval (PFI), and disease-specific survival (DSS); however, multivariate analysis identified only pTNM staging as an independent factor, suggesting that the small sample size and confounding may obscure its independent predictive value (91). In esophageal adenocarcinoma (EAC), a 6-gene model incorporating *ESM1* can predict OS, but it lacks functional experiments and independent clinical validation (128). Moreover, most existing studies have not distinguished endothelial-derived ESM1 from tumor cell-derived ESM1; circulating ESM1 may also be affected by non-tumor factors such as infection, bone marrow regeneration, and endothelial injury, and its glycosylation status may influence its function. Therefore, the specificity of ESM1 as a prognostic biomarker still requires validation in standardized, source-defined, prospective cohorts. Furthermore, no ESM1-targeted therapy has yet been tested in clinical trials, so these prognostic associations should not be interpreted as evidence for immediate clinical translation.

In gynecological and urogenital cancers, the prognostic value of ESM1 has also been reported. In OC, ESM1 is significantly upregulated in tumor tissues, and its expression is associated with poorer OS and progression-free survival (PFS); protein expression is associated with international federation of gynecology and obstetrics (FIGO) stage, lymph node metastasis, and recurrence (21). A prospective observational cohort study involving 100 patients with epithelial OC showed that ESM1 microvessel density was significantly associated with tumor histological type, size, stage, and grade, and was confirmed by multivariate analysis to be an independent prognostic marker for OS (129). In addition, *ESM1* was incorporated into a 15-gene extracellular matrix-related model selected using random forest and Lasso algorithms; this model was validated in the TCGA Pan-Cancer dataset and can be used for prognosis prediction and evaluation of immunotherapy efficacy in OC (130). Similarly, because *ESM1* is only one component of this 15-gene signature, its independent predictive value cannot be isolated from the model, and the observed performance reflects the combined effect of all genes rather than *ESM1* alone. However, most of these studies are based on database mining or single-center samples and lack standardized detection criteria and large-scale prospective validation. In BC, high ESM1 expression was associated with higher grade, more advanced stage, and poorer survival across three independent cohorts—METABRIC, SCAN-B, and TCGA-BRCA—and was confirmed as an independent adverse prognostic factor. This association was experimentally supported in a 4T1.2 mouse implantation-resection metastasis model, constituting one of the few existing lines of prognostic evidence for ESM1 that have been independently validated across multiple cohorts (131). In cervical cancer, a hypoxia-angiogenesis-related six-gene prognostic model incorporating *ESM1*, constructed based on TCGA and Gene Expression Omnibus (GEO) data, showed that the high-risk group had poorer OS (HR = 4.63, 95% CI not reported, P < 0.001); the risk score was an independent prognostic factor and was reproduced in the external GSE52903 cohort (132). However, *ESM1* is only one component of this six-gene signature, and its independent contribution to prognosis cannot be distinguished from the other genes. Therefore, this model does not establish *ESM1* as an independent prognostic biomarker. Moreover, this model was derived entirely from transcriptomic bioinformatics analysis, lacks functional experiments, and was validated using only a single GEO dataset; moreover, the independent contribution of *ESM1* within the model was not separately validated. In renal cell carcinoma (RCC), a retrospective analysis of 153 patients (based on prospectively collected data) showed that the ESM1 immunoreactivity score increased with clinical stage and was associated with local invasion and metastasis in ccRCC; patients with high scores had lower 10-year OS and disease-free survival (DFS) (98). In prostate cancer, serum ESM1 ≥1.8 ng/mL has been reported as an independent predictor of biochemical progression-free survival after radical prostatectomy (HR = 2.44, 95% CI 1.78–3.23, P = 0.001). *ESM1* mRNA has also been repeatedly selected by machine learning models as a strong predictor of postoperative biochemical recurrence. However, in the prostate cancer cohort, serum ESM1 levels did not differ significantly from those in the control group (P = 0.122), suggesting that it is more likely a prognostic rather than a diagnostic indicator; moreover, the machine learning results were based on internal validation using a single dataset of 187 cases and still require external validation (97, 133).

In other solid tumors and hematologic malignancies, including BC, DLBCL, NPC, pancreatic neuroendocrine tumors (PNET), PA and acute leukemia, high ESM1 expression is frequently associated with increased aggressiveness, a higher risk of recurrence, or poor survival (8, 80, 134–138). However, the evidence is generally less robust than that for digestive system tumors and is mostly derived from retrospective, single-center, or bioinformatics-based inference; its independent prognostic value remains to be validated. In terms of multigene prognostic models, *ESM1* is frequently incorporated into prognostic models for multiple cancer types, such as OC and CRC (123, 130). However, such evidence merely indicates that *ESM1* is a component of these models and does not establish its independent prognostic value; therefore, this evidence should be considered supplementary reference rather than a core argument.

Expression levels of ESM1 in various tumors are closely associated with patient clinical outcomes, and meta-analyses and multiple retrospective cohort studies support its role as a potential prognostic biomarker. However, the vast majority of these studies are retrospective and single-center in design, lack external validation cohorts, and lack clear standards for ESM1 detection methods and threshold settings, making it difficult to directly compare results across studies. The sources of heterogeneity across these studies are multiple and systematic. They include small, single-center, retrospective cohorts with limited external validation; different detection platforms and assays (e.g., ELISA, IHC, RT-qPCR, single-cell RNA sequencing) with varying sensitivity and specificity; inconsistent cut-off values and units across studies; differences in sample types (serum, plasma, urine, tissue) and in the cellular source of ESM1 (endothelial vs. tumor vs. stromal); variable adjustment for confounders such as stage, treatment, and inflammation; and potential publication bias. These factors apply to both diagnostic and prognostic studies and should be taken into account when interpreting the reported AUC and HR values. Future efforts should focus on clinical translation, including conducting prospective, multicenter cohort studies and standardizing detection methods and thresholds. In addition, the number of studies on certain cancer types is limited; for example, there is only a single report on PNET, and the conclusions require further validation. At present, however, all ESM1-targeted strategies remain preclinical, and no clinical trial has yet been initiated; therefore, any clinical translation remains speculative and requires rigorous prospective validation.

### 5.3 Therapeutic value: efficacy monitoring, resistance prediction, and combination sensitization

In terms of dynamic therapeutic monitoring, ESM1 levels have been reported to reflect dynamic changes in tumor burden. In BC, preclinical models and a small-sample clinical study showed that serum ESM1 levels were positively correlated with tumor burden and decreased significantly after tumor resection; among 20 patients with postoperative recurrence and negative conventional biomarkers such as CEA and cancer antigen 15-3 (CA15-3), more than 60% were ESM1-positive (93). However, the study only assessed sensitivity in patients with recurrence and did not include a control population confirmed to be recurrence-free, so specificity has not been established; moreover, the enrolled patients with recurrence were not pre-screened for ESM1 expression, and approximately 25% of patients with recurrence were negative for all three markers, suggesting that ESM1 can only serve as a supplementary rather than a replacement marker. In patients with acute leukemia, serum and bone marrow ESM1 levels were highly synchronized with disease status: highly expressed at initial diagnosis, decreased after remission, and elevated again at relapse. If chemotherapy failed to achieve remission, the levels remained unchanged; ESM1 was also elevated during the bone marrow regeneration phase (8). Moreover, a study of AML treatment found that blood ESM1 levels were elevated regardless of treatment response (139), suggesting that its fluctuations are not specific to tumor progression. In low-grade glioma (only 19 patients), postoperative ESM1 levels first increased and then decreased; the early postoperative elevation was considered possibly related to the inflammatory response caused by surgery itself rather than residual tumor (100), this study had a small sample size and lacked long-term follow-up, making it impossible to assess its association with recurrence. Therefore, when interpreting dynamic changes in ESM1, a comprehensive assessment should be performed in conjunction with the patient’s clinical status to avoid misinterpreting benign elevations as tumor progression. However, these findings are based on small-sample or retrospective studies, and no ESM1-targeted monitoring strategy has yet entered clinical trials; therefore, their clinical utility remains speculative.

In terms of predicting treatment outcomes, no clinical study has directly validated the association between baseline ESM1 levels and the efficacy of targeted therapy, and all currently available evidence is derived from preclinical mechanistic studies. Serum ESM1 levels in patients with RCC are 3–10 times higher than those in healthy individuals, and *in vitro* experiments showed that VEGF-induced ESM1 release from endothelial cells can be blocked by the anti-angiogenic drug sunitinib (19). In BCa, tumor vascular *ESM1* expression is associated with stage and invasiveness and can predict shorter recurrence-free survival in non-invasive BCa (20). These findings only suggest that ESM1 may serve as a promising candidate marker for monitoring the response to anti-angiogenic therapy; however, its predictive value has never been validated in prospective treatment cohorts and should not be regarded as an established conclusion. Moreover, because no ESM1-targeted therapy has yet entered clinical trials, any clinical translation of these findings remains speculative and requires prospective validation.

In terms of mechanisms of chemotherapy resistance, multiple *in vitro* experiments have revealed the molecular mechanisms by which ESM1 promotes chemotherapy resistance. In OC, ESM1 mediates platinum resistance through activation of pathways such as Wnt/β-catenin and PI3K/AKT (36, 74). These mechanistic studies suggest that evaluating the expression levels of ESM1 and related molecules, such as lactate and SCD1, may help prospectively identify patients who are insensitive to platinum-based therapy; however, these studies are based on cell line experiments, and whether their conclusions can be extrapolated to patients remains to be determined through *in vivo* and clinical validation. These findings are preliminary and should not be interpreted as established mechanisms of clinical resistance.

As a molecular marker that dynamically reflects tumor burden and angiogenesis, ESM1 shows some potential in monitoring treatment response, particularly offering possible complementary value for patients with recurrence who test negative for conventional biomarkers. In terms of predicting treatment efficacy, the currently available evidence consists mainly of preclinical mechanistic studies, and its clinical predictive value still needs to be validated in prospective cohort studies. Future studies should prioritize the standardization of detection methods, conduct prospective validation trials, and explore strategies for combining ESM1 with other biomarkers to advance its clinical translation. At present, however, all ESM1-targeted strategies remain preclinical, and no clinical trial has yet been initiated; therefore, any clinical translation remains speculative and requires rigorous prospective validation.

### 5.4 ESM1 as a direct therapeutic target

In mouse tumor models induced by *ESM1* overexpression, a monoclonal antibody targeting the phenylalanine-rich (F) region of ESM1 has been reported to inhibit tumor growth in SCID mice (69). In addition, at the molecular structural level, the 50 amino acids encoded by exon 2 of *ESM1* are essential for its tumor-promoting function: the splice variant endocanΔ2, which lacks this exon, has been reported to lose its tumor-promoting capacity due to impaired glycan synthesis and altered multimerization (140).In lung cancer, *ESM1* mRNA was selectively elevated in tumor endothelial cells, whereas endocanΔ2 mRNA remained unchanged, suggesting relative enrichment of the tumor-promoting splice isoform (140). This may provide a rationale for developing drugs that specifically target the tumor-promoting domain (the F region/exon 2-encoded sequence) without interfering with the physiological functions of full-length ESM1. However, endocanΔ2 splicing appears to be primate-specific, and mouse models lack this isoform, which limits preclinical modeling (140). Moreover, no ESM1-targeted drug has yet entered clinical trials.

Certain drugs and natural products have been reported to downregulate ESM1 expression. High-throughput drug screening identified the natural anthraquinone compound aloe-emodin as targeting the ESM1 active pocket and has been reported to inhibit the ESM1/ZEB1/EMT signaling axis as well as cell proliferation and invasion in cervical cancer *in vitro* and in nude mice (59). Early studies in BCa suggest that interfering with the interaction between ESM1 and VEGFR-2/VEGF-A may be another strategy for inhibiting tumor angiogenesis (20). In addition, in nude mouse models, miR-9-3p derived from bone marrow mesenchymal stem cell exosomes has been reported to directly bind to and downregulate *ESM1* mRNA, thereby inhibiting the *in vivo* growth and metastasis of BCa (34). Although its specific mechanism of action remains unclear, this may provide a proof of concept for exploring exosome-based ESM1-targeted delivery strategies.

Research on ESM1 as a therapeutic target has expanded from gene knockdown to strategies such as antibody therapy, small-molecule compounds, and exosome delivery. However, all currently available evidence is derived from cell lines and animal models, and no ESM1-targeted drugs have entered clinical trials. These strategies remain entirely preclinical, and their safety, efficacy, and tumor selectivity have not been evaluated in humans. Therefore, clinical translation remains speculative and requires extensive validation. Future studies should focus on the development of ESM1 monoclonal antibodies and the precise targeting of tumor-promoting functional domains.

### 5.5 Combination strategies and sensitization to existing therapies

Preclinical evidence suggests that ESM1 may play a dual role in combination therapy: it may serve as a “synergistic target” to enhance the efficacy of existing therapies, or as a “sensitizing agent” to participate in reversing therapeutic resistance. However, the currently available evidence is mostly derived from preclinical models or retrospective clinical observations, and the clinical benefit of directly targeting ESM1 has not yet been confirmed in prospective trials. Moreover, no ESM1-targeted strategy has yet entered clinical trials, and any clinical translation remains speculative.

As a synergistic target, ESM1 inhibition in combination with anti-angiogenic therapy has been reported to exhibit synergistic effects in preclinical models of bevacizumab resistance. In preclinical models of bevacizumab-resistant human CRC and BC, resistant tumors highly expressed ESM1, and the combination of an anti-ESM1 monoclonal antibody with bevacizumab improved tumor response (27, 54). However, these findings are limited to preclinical models, and independent validation in clinical settings is still required.

As a sensitizing agent, ESM1 inhibition has been reported to enhance therapeutic response in some preclinical models. In prolactinoma, ESM1 is associated with resistance to dopamine (DA) agonists; *in vitro* silencing of *ESM1* not only inhibited tumor cell viability but also was reported to enhance sensitivity to DA treatment (23). In radioresistant BC, knockdown of *ESM1* effectively reversed the proliferative and migratory capacities of radioresistant cells and reduced tumor-promoting signaling, including MMP-9, VEGF-A, HIF-1α, NF‐κB, and STAT-3 (77). These findings suggest that ESM1 may serve as a sensitizing target for hormone therapy and radiotherapy; however, the study did not directly examine whether ESM1 inhibition restores radiosensitivity, and further *in vivo* and clinical studies are required to confirm this. No clinical trial has yet been initiated to test these combination strategies.

ESM1 may have dual value in combination therapy: targeting ESM1 may produce synergistic effects with anti-angiogenic therapy, and it may also serve as a sensitizing agent to enhance sensitivity to therapies such as dopamine agonists. Direct evidence for its synergistic effect with immune checkpoint therapy is currently lacking and requires further validation. The safety of combination therapy requires systematic evaluation, particularly because dual anti-angiogenic strategies may increase the risk of vascular-related toxicities such as bleeding and hypertension. In addition, the delivery efficiency and safety of ESM1-targeting strategies still need to be optimized, and no prospective clinical trial has yet confirmed that ESM1-based combination strategies can improve patient outcomes. Therefore, at present, these strategies remain entirely preclinical, and their clinical translation remains speculative. In the future, with the development of specific ESM1-targeted agents, ESM1-based sensitization and synergistic strategies may eventually provide new therapeutic options for patients with resistant tumors, but this requires extensive prospective validation.

## 6 Conclusion

ESM1 is a key molecular node integrating tumor angiogenesis, immune evasion, and therapeutic resistance, and its potential as a circulating biomarker and therapeutic target warrants further investigation. However, translation from molecular mechanisms to clinical application remains limited by several critical gaps. Most current evidence is preclinical or retrospective, and the strength of evidence is uneven: the pro-tumorigenic roles of ESM1 are supported by a substantial body of mechanistic and correlative studies, whereas the tumor-suppressive effects observed in prostate cancer, GC, CRC, and non-glycosylated ESM1 are preliminary, context-dependent, and far less robust. These opposing functions should not be regarded as equally established.

Several unresolved issues further constrain clinical translation. First, the cellular source of ESM1—endothelial, tumor, or stromal—has rarely been rigorously distinguished, yet it may critically affect both functional interpretation and biomarker performance. Moreover, the contradictory phenotypes across cancer types lack cross-cancer synthesis, and the key microenvironmental variables that switch ESM1 function remain poorly defined. Second, although glycosylation status has been proposed to determine the functional direction of ESM1, this remains a hypothesis; no study has directly manipulated or compared different glycosylation states in tumors or in the tumor microenvironment. Third, the predictive value of ESM1 for immune checkpoint blockade is inconsistent across cancer types, and its cross-cancer diagnostic value remains unproven because of non-standardized assays, variable cutoff values, small retrospective cohorts, and lack of head-to-head comparison with established biomarkers; in addition, in multi-gene models, the independent predictive contribution of *ESM1* cannot be separated from that of the other genes. Fourth, no prospective clinical trial has demonstrated clinical benefit from ESM1-targeted therapy, and the safety, toxicity, and feasibility of selectively targeting tumor-associated ESM1 while preserving normal endothelial function remain unknown.

Future research should therefore be explicitly designed to address these gaps (1). Mapping the expression regulatory networks of *ESM1* across cancer types and microenvironments, with explicit attention to tissue-specific versus universal inducers, would help define the cellular sources and context-dependent determinants that underlie its contradictory phenotypes and would facilitate cross-cancer synthesis (2). Determining how post-translational modifications, especially glycosylation, affect the functional switch between pro-tumor and anti-tumor forms, would directly test the currently speculative glycosylation balance hypothesis through direct experimental manipulation in tumor tissues and the tumor microenvironment (3). Establishing standardized detection protocols and cutoff values for multiple body fluids, followed by prospective, multicenter validation with inflammatory controls and comparison against existing biomarkers, would address the heterogeneity of current diagnostic and prognostic evidence, clarify the independent contribution of *ESM1* in multi-gene models, and help resolve its inconsistent predictive value for immune checkpoint blockade (4). Developing tumor-selective ESM1-targeting strategies and evaluating them in combination with anti-angiogenic, immune checkpoint, and metabolic therapies, with systematic safety assessment. If these basic and clinical gaps can be addressed, ESM1 may eventually serve as an integrated biomarker and therapeutic target with diagnostic, prognostic, and therapeutic value. At present, however, its clinical translation remains exploratory and requires rigorous prospective validation.
